# Physical activity and quality of life in children: Findings from the Health Oriented Pedagogical Project (HOPP)

**DOI:** 10.1371/journal.pone.0353686

**Published:** 2026-07-10

**Authors:** Rein Magnus Jensen, Asgeir Mamen, Christoffer Wang, Per Morten Fredriksen

**Affiliations:** 1 Faculty of Education and International Studies, Oslo Metropolitan University, Oslo, Norway; 2 Faculty of Health and Technology, Department of Health and Exercise, Kristiania University College, Oslo, Norway; 3 University of Inland Norway, Faculty of Social and Health Sciences, Department of Public Health and Sport Sciences, Section for Public Health, Norway; All India Institute of Medical Sciences, INDIA

## Abstract

**Purpose:**

The purpose of this study was to examine the associations between a 5-year school-based physical activity (PA) intervention and quality of life (QoL) in children aged 6–12 years.

**Methods:**

Data were collected through the Health Oriented Pedagogical Project (HOPP), a longitudinal study conducted in Norway. HOPP involved children and their parents from nine elementary schools (n = 2,140 children and 1,639 parents completed the QoL-questionnaire). Seven schools received the intervention (an additional 225 minutes of physical activity per week), while two schools served as controls following the standard curriculum. QoL was measured using the Inventory of Life Quality in Children and Adolescents (ILC), and physical activity (PA) was assessed using accelerometers. Covariates included father’s education level as a proxy for socioeconomic status (SES) and children’s waist-to-height ratio (WHtR).

**Results:**

The analysis revealed a significant positive association between MVPA and QoL (β = 0.008, *p* < 0.001), although the effect size was small. SES was significantly associated with QoL in intervention schools (β = 0.249, *p* < 0.001), while WHtR was negatively associated with QoL in both groups, with a stronger association in control schools (β = −4.344, *p* = 0.010). The control schools exhibited higher QoL scores than the intervention schools, with an average 0.5-point advantage (β = 0.458, *p* < 0.001), likely reflecting underlying SES differences.

**Conclusion:**

This study highlights the complex interplay between MVPA, SES, WHtR, and QoL in children. While MVPA was associated with better QoL, these associations varied according to factors such as age, SES, and WHtR, with no significant association observed for sex. The findings suggest that sustained and varied physical engagement in school settings may be relevant for children’s QoL. Moreover, school-based initiatives should consider multiple individual and environmental factors, particularly SES and physical health metrics, when interpreting or targeting QoL-related outcomes.

**Trial registration:**

The study is registered at ClinicalTrials.gov (Identifier: NCT02495714). The trial was retrospectively registered on June 20, 2015. Baseline data collection was initiated in mid-January 2015.

## Introduction

Identifying and addressing negative societal trends is essential for initiating necessary interventions and implementing countermeasures. Shifts in a population’s quality of life hold significant importance for various stakeholders, including health authorities, policymakers, school counselors, child and family therapists, and parents [1]. To understand what affects children’s well-being over time, it is essential to conduct epidemiological studies within cohorts of healthy children [2].

The World Health Organization (WHO) defines Quality of Life (QoL) as an individual’s perception of their position in life in the context of the culture and value systems in which they live and in relation to their goals, expectations, standards, and concerns [3]. QoL is a broad and multifaceted term encompassing various aspects of life, such as physical, social, and emotional dimensions [4]. QoL can be measured subjectively, on the basis of individual evaluations, or objectively, based on measurable factors such as physical fitness and private economy [2]. For example, studies indicate that children from low-income families tend to have lower QoL scores compared to children from high-income families [5,6]. Furthermore, the baseline results from the Health Oriented Pedagogical Project (HOPP) revealed a strong connection between parents’ educational level and QoL [7], which aligns with previous research [8], and is used in this study as a proxy for SES.

In Norway, reports from the Norwegian Social Research Institute (NOVA) indicate that approximately 90% of children and adolescents aged 10–12 years report being satisfied with life [9,10]. Norwegian studies have shown a stable or slight decrease in satisfaction with life from childhood to adolescence, and in general, girls (15%) are less satisfied than boys (10%) [9,10]. International studies have also revealed a decrease in QoL with increasing age in adolescents [11–13].

### QoL and ILC

Health is a central component of QoL and has consistently been associated with both physical health and mental health outcomes [14] and is therefore often conceptualized as health-related quality of life (HRQoL) [15,16]. The introduction of HRQoL marked a shift from looking at health and well-being as the absence of illness to a broader perspective that considers factors that provide value and meaning [2].

In recent decades interest in developing multidimensional QoL instruments for children and adolescents that cover not only physical functioning, but also mental health has increased [17]. Both are assessed in the standardized Inventory of Life Quality in Children and Adolescents (ILC) [18,19]. The ILC inventory was originally developed for children with mental or physical health problems, but it has since been adapted to the general population, and may be used to investigate results across decades [20]. The ILC inventory addresses the child’s subjective experience of their life situation, with particular emphasis on health condition, general functioning, social integration, and participation in age-specific activities [21]. Specifically, ILC assesses perceived QoL in general, and six items address physical and mental health, family and school functioning, social contact with peers, interests and recreational activities. ILC also comes with a corresponding parental version [22].

### QoL, physical activity and health

The association between physical activity (PA) and quality of life (QoL) among children and adolescents is well-documented. A comprehensive meta-analysis reported a small but significant effect size for PA interventions on QoL, with Cohen’s d = 0.173 (95% CI: 0.106–0.239, p < 0.001), with stronger effects observed among adolescents than younger children [23]. International guidelines also recommend regular physical activity for overall health and well-being [24]. PA has been shown to reduce mental health issues such as depression and anxiety while promoting prosocial behavior [25,26].

An Australian study demonstrated that children maintaining high PA levels and low screen time achieved significantly higher QoL compared to those with low PA and high screen time [5]. Similarly, baseline data from the HOPP study highlighted that aerobic fitness positively influenced QoL [7].

Findings from the Tromsø study (Fit Futures) further supported the role of aerobic fitness in enhancing QoL. For every additional 300 meters covered in an endurance test, QoL increased by 3 points on the scale [23]. These results emphasize the central role of physical fitness in supporting well-being among children and adolescents. Research suggests that psychological well-being in early adolescence can predict PA levels two years later [30], and meeting PA recommendations of over 60 minutes of moderate-to-vigorous activity a day is associated with better self-reported QoL [31,32].

Issues concerning body mass are not limited to adults or adolescents. A Californian study found that concerns about weight and body dissatisfaction are highly prevalent among third-grade girls and boys, across ethnicity and socioeconomic factors [33]. Research has also shown that overweight or obese individuals typically experience *lower QoL.* A study involving 576 adolescents found that vigorous PA, cardiorespiratory fitness, and BMI were related to higher scores on QoL while BMI influenced QoL indirectly through its effects on depressive symptoms and cardiorespiratory fitness [34]. The baseline data from the HOPP study, concluded that BMI and waist circumference were negatively associated with QoL [7].

Research on PA levels among Norwegian children and adolescents shows that 74% of children aged 10–12 years engage in sports as a leisure activity, with a slight male predominance [9]. Among adolescents, 79% report exercising more than once a week [10]. Research indicates age-related variations in PA, revealing a decline from early school years to secondary school, and further into high school [10,35,36].

The purpose of this study was to *examine the association between* a 5-year school-based PA intervention and QoL in children aged 6–12 years.

## Methods

### Study design

The data for the present study were collected as part of the larger HOPP study, a longitudinal case-control intervention designed to examine the association between increased PA within a broader pedagogical framework focusing on both health and academic outcomes [37]. The primary aim was to integrate increased PA into the educational curriculum, targeting children from 1st to 6th grade in elementary schools in Norway, particularly in the Horten municipality and the Oslo metropolitan area.

The intervention was inclusive, cost-effective, and designed to reach children across all socioeconomic backgrounds by utilizing existing school facilities and teacher resources. The intervention emphasized mastery and confidence by transforming passive, desk-based learning into active, movement-based activities designed to enhance both academic skills and health. The intervention included approximately 45 minutes of additional PA per school day (225 minutes per week), replacing traditional desk-based learning with curriculum-aligned activities in language, mathematics, and English. These activities, tailored for each grade (1–7), were organized into grade-specific “activity boxes,” allowing teachers to seamlessly integrate PA into daily learning. Combined with the standard 90 minutes of physical education per week, the intervention provided a total of 315 minutes of weekly PA. Teachers reported daily on the duration and intensity of activities at the class level, but individual participation was not monitored. Previous analyses from the HOPP project based on accelerometer data indicate that children accumulate approximately 28–32 minutes of MVPA during school hours per day, corresponding to only 17–19% of total daily physical activity [38]. Previous analyses from the HOPP study have shown that the intervention was associated with higher school-time MVPA compared to control schools, although the magnitude of the increase varied between classes [36]. This suggests that school time represents a relatively low-activity domain. The HOPP intervention was designed to address this by introducing an additional 45 minutes of daily physical activity, thereby representing a substantial potential increase in school-based physical activity exposure. However, individual-level compliance and variation in participation may have influenced the actual magnitude of this increase.

Participation in the study required written parental consent, and children could withdraw at any time without providing an explanation. Data collection commenced in spring 2015 and continued until participants completed primary school in 2020. A detailed study protocol, including the library of activities, is available [37].

### Population

Parents and children aged 6–12 years, from nine elementary schools were invited to participate. Seven primary schools in Horten municipality were designated as intervention schools, while the two control schools were located near the Oslo metropolitan area. Both regions were predominantly upper-middle-class, but the control schools were located in areas with comparatively higher socioeconomic status. In 2015, informed consent was obtained from parents of 2,297 children, representing 82% of the target population of 2,816. Among these participants, 2,140 children (93%) and 1,639 parents (71%) completed the QoL-questionnaire.

### Data collection

Measurements of PA have often been questionnaire-based and retrospective in nature [27], which may be vulnerable to recall bias [39]. While questionnaire-based measures that have undergone rigorous validation are considered valid, sensitive, and reliable, they are not without limitations. Similarly, wearable devices such as accelerometers, despite being valuable tools for objective measurement, also come with their own set of limitations and biases, such as over- or under-estimation of certain movement behaviors, particularly in young children, depending on the wear protocol and data processing [27,39]. A combination of questionnaires and wearable devices would have been preferable; however, in the present study, accelerometers were used to assess physical activity.

### Inventory of Life Quality in Children and Adolescents (ILC)

The primary outcome in this study was the QoL score (LQ0–28) derived from the ILC instrument [21], which was used consistently in all analyses. The ILC assesses a global quality of life (QoL) score and measures seven key life areas: school performance, family relations, peer relations, autonomy in play, physical health, mental health, and a global assessment of well-being.

Responses to each item are rated on a five-point Likert scale, ranging from 1 (no problem) to 5 (severe problem). The instrument generates scores for problematic life areas and overall quality of life, reflecting both the presence of problems (problem score) and a positive assessment of QoL across all seven areas [21]. The instrument has demonstrated acceptable reliability and validity in previous international studies [18]. The Norwegian version of the ILC has been translated and psychometrically validated in samples of primary school children and their parents, with reported satisfactory construct validity [21] and test–retest reliability [41,42].

### Procedure

Parents were provided with a digitized version of the ILC questionnaire and informed consent through email or satchel post. Parents were asked to provide information about their educational level, age, gender, and relations to the child. The questionnaire was designed to ensure anonymity, and unique identification numbers were used for data collection.

For children, the questionnaire was completed during school hours, under supervision for younger pupils. Fourth to sixth-grade students were able to complete the form without assistance. ILC was completed anonymously using digital methods, with data securely transferred to the database during annual testing.

### Physical activity

To assess PA, an activity monitor accelerometer (ActiGraph wGT3X-BT, ActiGraph LLC, Pensacola, FL, USA) was used. The device was attached to the right side of the child’s hip with an elastic band for seven consecutive days, at all hours, unless injured, ill, showering, swimming, or absent from school. The sampling frequency was set to 100 Hz at 10 s epochs. A minimum of 8 hrs/day for one day of registered PA was required for data analysis. Nonwear time was excluded using the Troiano technique with 60 min of consecutive zeroes and a tolerance of 2 min of activity [40]. Valid hours were defined as 06:00–23:59 in ActiLife 6 (ActiGraph LLC, Pensacola, Florida, USA).

Categorical division of PA levels was based on mean counts per minute (cpm) as sedentary (0–99 cpm), light (100–1999 cpm), moderate (2000–4999 cpm), and vigorous (≥ 5000 cpm), and the number of minutes in each intensity domain was recorded [40]. Moderate-to-vigorous physical activity (MVPA) was calculated by summing minutes in moderate and vigorous intensity domains divided by the number of valid days.

### Ethical considerations

Informed consent was obtained from all parents before their inclusion. Approval for the study was granted by the Regional Committee for Medical and Health Research Ethics (reference number 2014/2064/REK sør-øst), ensuring adherence to ethical standards in research. The study is registered as a clinical trial (ClinicalTrials.gov Identifier: NCT02495714). This analysis utilizes data from January 15^th^, 2015, to May 15^th^, 2020. The study was conducted in accordance with the Declaration of Helsinki.

### Statistical analysis

ILC responses were aggregated into two scores based on the Norwegian manual provided by the ILC Nordic distributor, Hogrefe AB [41,42]. These scores are the problem score (PR0–7) and the QoL score (LQ0–100). The PR0–7 score is computed by dichotomizing responses to each of the seven items, where ratings of 1 or 2 are coded as 0 (no problems) and ratings of 3, 4, or 5 are coded as 1 (problem present). The LQ0–28 scale is derived from the PB35 raw score (the sum of all seven items rated on a scale of 1–5), with higher LQ0–28 values indicating better quality of life. All statistical analyses were conducted using the QoL score (LQ0–28) as the primary outcome variable.

The raw total score ranges from 7 (minimum, all responses rated as 1) to 35 (maximum, all responses rated as 5). Subtracting the lowest possible raw score (7) from the highest (35) gives a range of 28, which forms the basis for the LQ0–28 scale. To calculate the QoL score (LQ0–100): Subtract the PR0–7 score from the maximum score of 7 to calculate the LQ0–7 score, reflecting the absence of problems. Convert the LQ0–7 score to a percentage by dividing it by 7 and multiplying by 100, yielding the LQ0–100 score. Higher LQ0–100 values indicate better quality of life. An example score of 24 on the LQ0–28 scale corresponds to 85.7% on the LQ0–100 scale. A percentage of 85.7 reflects high QoL. The LQ0–100 score was used for descriptive purposes only and was not included in the main mixed model analyses.

The dataset includes baseline, secular and longitudinal observations within the same analytical framework. Linear mixed models were used to account for repeated measures within individuals over time. Alternative repeated covariance structures were evaluated using Akaike’s Information Criterion (AIC) within the same mixed-model specification. Diagonal and compound symmetry structures were compared for both child- and parent-reported QoL outcomes, and the diagonal structure was retained based on the lowest AIC. As the pupils from the 1^st^ grade in 2015 have repeated the test in all years through to 2020, they have 5 years longitudinal follow-up. Similar for the 2^nd^ year pupils in 2015, as they have 4 years longitudinal follow-up, and so forth for pupils at older age in 2015. At the same time the model includes all the data for each year, hence we also have secular trends with reduced number of pupils for each year of intervention.

ANOVA was used to examine baseline differences in QoL score (LQ0–28) by grade and school group (intervention/control) for both child- and parent-reported data. A backward variable selection procedure was applied within the linear mixed model framework, with child-reported QoL (LQ0–28) as the dependent variable. Candidate fixed effects included sex, MVPA, test year, SES, WHtR, and school group. Analyses were conducted using available data. Missing data were handled using complete-case analyses within the mixed model framework, with no imputation performed. No formal sensitivity analyses for missing data were conducted. Due to the longitudinal design, participant numbers varied across time points as cohorts entered and exited the study.

In Norway, socioeconomic status is commonly determined by parental education, with higher levels of education corresponding to higher SES. Parental education reflects both economic resources and social capital within households and is widely used as a proxy indicator in population-based studies [7]. In this study, SES was assessed using fathers’ education level. Educational attainment was categorized as primary and secondary school (SES = 1), high school (SES = 2), bachelor’s degree (SES = 3), and master’s degree or higher (SES = 4). Sensitivity analyses using alternative SES indicators, including maternal and highest parental education, produced similar results. Fathers’ education showed the most consistent association with QoL and was therefore retained as the primary SES indicator in the main analyses.

Analyses were conducted with the Statistical Package for the Social Sciences (SPSS) version 28 (IBM, Armonk, NY, USA) and Number Cruncher Statistical System (NCSS) 24.0.7 (LLC, Kaysville, UT, USA). An-value of 0.05 was used for statistical significance.

## Results

### Baseline

**Fig 1:**
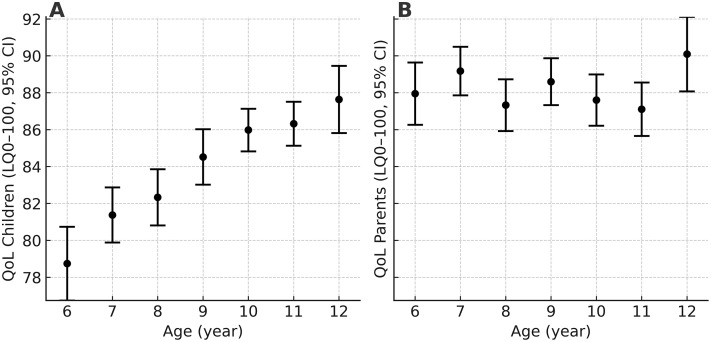
Baseline QoL scores for children (A) and parents by proxy (B) across age groups. Children scored themselves lower than parents by proxy. The y-axis was narrowed to better visualize small differences in QoL (approximately 77–92).

Cross-sectional measurements in children using ANOVA at baseline show an increase in QoL with increasing age (*p < 0.001*), as shown in Fig 1. However, the response from parents by proxy illustrates a nonsignificant change across age.

In Table 1, the years of testing are displayed in the left column. The upper horizontal row reveals dual information: ^1)^ the cohort divided from 1^st^ – 6^th^ grade for the baseline values, and ^2)^ the number of years the pupils participated in the study (1–6 years) for *longitudinal* and *secular trends*. Baseline results are revealed along the 2015 horizontal upper row. Secular trends are displayed in the columns under each year in the study (1–6 years). The longitudinal results are presented along the diagonal axis from the 1^st^ results in 2015 to the last result in 2020. Results are presented separately for intervention and control schools.

**Table 1:** The results of Quality-of-Life data (LQ0-28) for the whole sample, divided into grades and intervention/control schools. The baseline data are listed horizontally along the year 2015. The longitudinal data are marked in light grey. The secular trends are listed at the bottom of each table. Cohen’s d is given in the last row.

	Grades												
	1		2		3		4		5		6		
Intervention	Mean	SD	Mean	SD	Mean	SD	Mean	SD	Mean	SD	Mean	SD	
**2015**	22.76	3.39	22.77	3.24	22.96	3.14	22.45	5.44	23.57	2.67	23.62	2.99	**Baseline**
**2016**			22.20	4.10	22.88	3.75	23.16	3.75	23.13	3.08	23.76	2.81	
**2017**					22.91	3.28	23.33	3.37	23.50	2.61	23.52	3.04	
**2018**							23.02	3.58	23.46	3.25	23.47	2.81	
**2019**									23.17	2.98	23.22	2.87	
**2020**											23.18	3.09	**Longitudinal**
	22.76	3.39	22.49	3.67	22.92	3.39	22.99	4.04	23.37	2.92	23.46	2.94	**Secular**
**Control**													
**2015**	22.29	3.72	22.87	3.33	24.01	2.64	24.46	2.31	24.63	2.48	24.61	2.68	**Baseline**
**2016**			22.59	3.53	22.63	3.67	24.44	3.02	24.44	2.48	24.47	2.77	
**2017**					23.41	3.30	23.73	3.24	24.14	2.95	24.75	2.84	
**2018**							23.62	2.54	23.84	3.13	24.72	2.40	
**2019**									24.25	2.95	23.92	2.56	
**2020**											23.94	2.8	**Longitudinal**
	22.29	3.72	22.73	3.43	23.35	3.20	24.06	2.78	24.26	2.80	24.4	2.68	**Secular**
**Cohen’s *d***													
**Intervention vs Control**	−0.04		−0.07		−0.13		−0.31		−0.31		−0.33		

**Fig 2:**
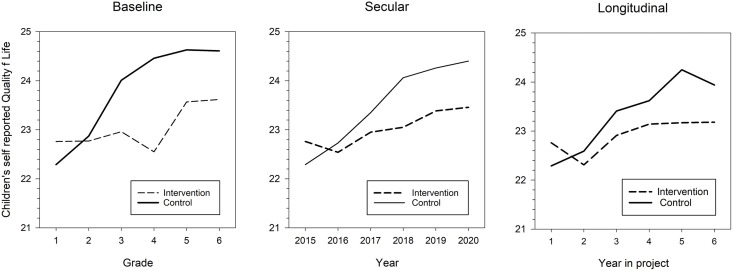
The figures show the crude values of QoL (LQ0–28) for children at baseline (left), secular trends (middle), and longitudinal trends (right). The Y-axis was narrowed (21–25) to better show the differences, and the X-axis shows grade (baseline), number of years in the study (longitudinal), or the year the data were accumulated (secular).

To illustrate the difference between baseline, secular and longitudinal trends, results are presented in three graphs (Fig 2), separated by intervention and control schools. The first graph of baseline measures uses grades 1–6 (6–11 years) on the X-axis. The longitudinal dataset displays the number of years the children have been involved in the study on the X-axis. For secular trends the accumulation at each year of the study is displayed by 2015–2020 on the X-axis. To emphasize variations in the data, as the data is narrow relative to the absolute values, the Y-axis was narrowed to better visualize small differences.

### Mixed model analyses

The diagonal covariance structure provided the best model fit across all analyses and was therefore retained. The differences in AIC between the tested structures were small for parent-reported outcomes (ΔAIC = 2.00) and somewhat larger for child-reported outcomes (ΔAIC = 4.29), but the overall pattern was consistent.

The mixed model analyses revealed significant associations between QoL and group (control vs. intervention), MVPA, WHtR, and parental education (Table 2), while no significant association was observed for sex. The control schools had higher QoL scores than the intervention schools by almost half a point (β = 0.458, p < 0.001). Similarly, parental education (father) showed that on a four-level educational scale—1) primary/secondary school, 2) high school, 3) bachelor’s degree, and 4) master’s degree or higher—QoL increased by approximately 0.2 points for each educational level (β = 0.204, p < 0.001).

**Table 2:** Linear mixed model results from the backward variable selection procedure*.

Model	Variable	B	SE	β	t	*p*-value
**1**	**Constant**	−247.99	62.336		−3.978	<0.001
	**Sex**	−0.32	0.090	−0.005	−0.351	0.725
	**Test year**	0.135	0.31	0.064	4.358	<0.001
	**WHtR**	−3.206	0.931	−0.050	−3.443	<0.001
	**AMVPA**	0.006	0.002	0.055	3.559	<0.001
	**SES**	0.204	0.055	0.057	3.697	<0.001
	**Control vs intervention**	0.461	0.099	0.073	4.647	<0.001
**2**	**Constant**	−248.73	62.295		−3.993	<0.001
	**Test year**	0.135	0.031	0.064	4.372	<0.001
	**WHtR**	−3.183	0.929	−0.050	−3.427	<0.001
	**AMVPA**	0.006	0.002	0.057	3.754	<0.001
	**SES**	0.204	0.055	0.056	3.689	<0.001
	**Control vs intervention**	0.458	0.099	0.073	4.634	<0.001

* Dependent variable: Quality of life (QoL; LQ0–28). Candidate fixed effects included sex, test year, waist-to-height ratio (WHtR), average moderate-to-vigorous physical activity (AMVPA), socioeconomic status (SES), and group (control vs. intervention). The backward-selected model retained test year, WHtR, AMVPA, SES, and group.

For MVPA, the association was small but statistically significant (β = 0.006, p < 0.001), corresponding to an increase of approximately 0.06 QoL points per additional 10 minutes of MVPA per day, or 0.36 points per additional 60 minutes. Test year was included as a proxy for duration of exposure to the intervention. The year 2015 represents baseline values, and each subsequent year until 2020 indicates one additional year in the intervention. Thus, 2020 represents five years of intervention exposure. In the present model, a significant association was observed for years in intervention, with an increase of 0.133 QoL points per year (β = 0.133, p < 0.001), corresponding to a total increase of approximately 0.665 points over five years. The largest association was observed for WHtR (β = −3.183, p < 0.001), corresponding to a reduction of approximately 0.32 QoL points per 0.1 increase in WHtR.

### Intervention/control

Linear mixed model analyses indicated that higher parental education (β = 0.249, *p < 0.001*) was associated with higher QoL among children in the intervention group (Table 3). Similar associations were observed for MVPA (β = 0.008, p < 0.001) and years in intervention (β = 0.136, p < 0.001), with higher levels of physical activity and longer participation associated with higher QoL, although the magnitude of the MVPA effect was small. WHtR was negatively associated with QoL (β = −2.681, p = 0.017). Sex was not significantly associated with QoL (β = −0.103, p = 0.392).

**Table 3:** Results from the linear mixed models for intervention and control schools *.

	Model	Variable	B	SE	β	t	*p*-value
**Intervention**	**1**	**Constant**	−242.75	80.204		−3.027	0.002
		**Sex**	−0.103	0.120	−0.017	−0.857	0.392
		**Test year**	0.132	0.040	0.063	3.324	<0.001
		**WHtR**	−2.754	1.125	−0.046	−2.448	0.014
		**AMVPA**	0.008	0.002	0.069	3.478	<0.001
		**SES**	0.248	0.073	0.063	3.403	<0.001
	**2**	**Constant**	−246.67	80.070		−3.081	0.002
		**Test year**	0.134	0.040	0.064	3.375	<0.001
		**WHtR**	−2.681	1.122	−0.044	−2.389	0.017
		**AMVPA**	0.008	0.002	0.074	3.901	<0.001
		**SES**	0.249	0.073	0.063	3.423	<0.001
**Control**	**1**	**Constant**	−277.67	99.717		−2.783	0.005
		**Sex**	0.116	0.138	0.020	0.845	0.389
		**Test year**	0.150	0.049	0.071	3.035	0.002
		**WHtR**	−4.086	1.698	−0.056	−2.406	0.016
		**AMVPA**	0.003	0.002	0.031	1.303	0.193
		**SES**	0.128	0.085	0.035	1.512	0.131
	**2**	**Constant**	−276.62	99.703		−2.774	0.006
		**Test year**	0.150	0.049	0.071	3.029	0.002
		**WHtR**	−4.190	1.693	−0.057	−2.475	0.013
		**AMVPA**	0.003	0.002	0.027	1.179	0.239
		**SES**	0.136	0.084	0.037	1.609	0.108
	**3**	**Constant**	−259.81	98.689		−2.633	0.009
		**Test year**	0.142	0.049	0.067	2.891	0.004
		**WHtR**	−4.199	1.694	−0.057	−2.479	0.013
		**SES**	0.137	0.084	0.037	1.622	0.105
	**4**	**Constant**	−266.74	98.639		−2.704	0.007
		**Test year**	0.145	0.049	0.068	2.968	0.003
		**WHtR**	−4.344	1.692	−0.059	−2.567	0.010

* Dependent variable: Quality of life (QoL; LQ0–28). Separate backward-selected linear mixed models were estimated for intervention and control schools. Initial models included sex, test year, waist-to-height ratio (WHtR), average moderate-to-vigorous physical activity (AMVPA), and socioeconomic status (SES). The final retained predictors are shown in the last model for each group.

In the control group, SES was not significantly associated with QoL (β = 0.137, p = 0.105). However, *duration of study participation* (β = 0.145, p = 0.003) and WHtR (β = −4.344, p = 0.010) were significantly associated with QoL.

### Socioeconomic status (SES)

**Fig 3:**
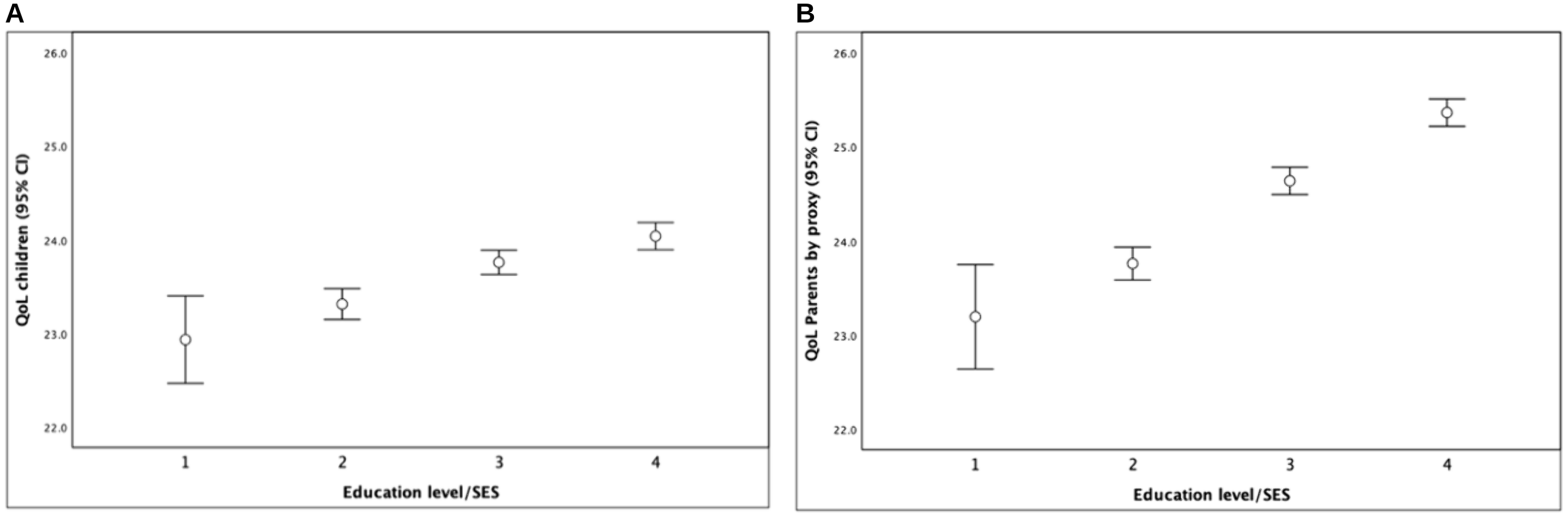
The association between SES and children’s QoL. Children’s responses are shown on the left and parent proxy reports on the right. Education level (SES) was categorized as: primary/secondary school = 1, high school = 2, bachelor’s degree = 3, and master’s/PhD = 4.

Fathers’ education, used as a proxy for SES, was positively and significantly associated with children’s QoL (Fig 3a-b). Both child- and parent-reported data showed a clear positive association between SES and QoL.

## Discussion

The purpose of this study was to examine the associations between a 5-year school-based physical activity (PA) intervention and quality of life (QoL) in children aged 6–12 years (grade 1 corresponds to approximately 6–7 years of age, while grade 6 corresponds to 11–12 years). Our findings indicate that while PA, measured as MVPA, was generally associated with better QoL, this association varied according to age, sex, WHtR, and SES. This suggests that the association between PA and QoL is shaped by a combination of individual and environmental factors.

The results revealed a small but consistent improvement in QoL with age for both the intervention and control groups. This contrasts with previous cross-sectional studies that have shown either a decline in QoL with increasing age or relative stability during childhood and adolescence [9–13].

### New findings in light of baseline data

Our longitudinal data provide insights into the trajectory of QoL in relation to PA. While baseline assessments identified aerobic fitness and muscular strength as significant contributors to QoL, our extended analysis suggests that the observed association between PA and QoL although statistically significant, is subtler than initially anticipated. This finding aligns with the baseline observation that not all physical attributes correlate directly with QoL improvements [7].

The findings indicate that the duration of participation in the intervention was associated with a small but cumulative increase in QoL. Each additional year in the intervention was associated with an increase in QoL culminating in a total increase of 0.7 points on the QoL-scale over a five-year period. However, given the narrow range of the scale and the high baseline values, this change is modest and of uncertain clinical relevance. This result may indicate that sustained engagement in school-based physical activity is associated with small, gradual increases in QoL over time. Notably, sex was not significantly associated with QoL scores, suggesting that the intervention was associated with similar outcomes across genders [9].

### Mixed model analyses

The mixed model analyses indicated that MVPA, WHtR, and SES were significantly associated with QoL. Parental education (father) was also significantly associated with QoL, with an increase of approximately 0.2 points in QoL for each step on a four-level educational scale (primary/secondary school, high school, bachelor’s degree, and master’s degree or higher).

MVPA was positively associated with QoL, although the magnitude of this association was small. An increase of 60 minutes of daily MVPA corresponded to an increase of approximately 0.36 points on the LQ0–28 scale, indicating that the association is modest relative to the total scale range. This is consistent with previous research reporting small effect sizes for the association between physical activity and QoL in children and adolescents [28,29].

While PA is often positively associated with health-related quality of life (HRQoL), the magnitude of this relationship tends to be modest and varies across populations [23]. Some studies have also reported no significant changes in mental health outcomes with variations in PA levels among adolescents, suggesting that other factors, such as psychosocial support or school environments, also play important roles [29]. Baseline findings from the HOPP study similarly indicated a positive association between physical fitness and QoL [7].

Our findings add to this body of work by suggesting that the association between physical activity and QoL is complex and limited in magnitude. While meeting recommended levels of MVPA (e.g., 60 minutes per day) has been associated with higher QoL in previous studies [31], the additional 225 minutes of weekly physical activity in the present intervention (approximately 45 minutes per school day) was associated with only small differences in QoL. This may reflect variability in intensity, individual engagement, or effective exposure to MVPA. Overall, these findings highlight the importance of sustained and context-sensitive physical activity engagement in relation to QoL, as also suggested in previous research [27].

### Intervention/control

Our analysis revealed differences between the intervention and control groups, providing insights into associations with QoL across different schools. In the intervention schools, SES, MVPA, years of participation in the intervention, and WHtR were all significantly associated with QoL.

In the control schools, SES was not associated with QoL, in contrast to the intervention schools, suggesting that the association between SES and QoL differed between the groups. However, although SES was included in the regression models, residual confounding related to school-level or contextual differences cannot be ruled out. In control schools, years of study participation and WHtR remained significant predictors of QoL. The consistent association of WHtR across both groups highlights the role of physical health indicators in QoL.

This difference should be interpreted with caution. Schools were not randomly assigned, and control schools were located in areas with generally higher socioeconomic status, increasing the risk of confounding. Although SES was included as a covariate, residual school- or context-level confounding may remain. Therefore, the group variable should not be interpreted as causal effects of the intervention.

Sex was not significantly associated with QoL, suggesting similar patterns across boys and girls. This aligns with previous Norwegian studies reporting limited gender differences in well-being among children aged 10–12 years [10]. While gender disparities in psychosocial factors like self-esteem and stress begin to emerge during late childhood, these trends are more pronounced during adolescence [9,10].

### Socioeconomic status (SES)

The results suggest that while SES is a crucial factor in the intervention group, it does not play a similar role in the control groups. In control schools, QoL appeared to be more strongly associated with duration of study participation and WHtR than with SES. As previously stated, the differences in outcomes between the control and intervention schools may partly be attributed to differences in SES between the two cohorts. Intervention schools were situated in a municipality known for lower SES, whereas the control schools were located closer to urban Oslo and likely represented a population with higher parental education levels.

This structural difference between school contexts may partly explain the higher QoL observed in control schools, rather than reflecting an absence of intervention effects. These findings are consistent with previous research highlighting the importance of socioeconomic factors for children’s health outcomes [5].

Although SES was significantly associated with QoL, the magnitude of this association was modest. A shift from the lowest to the highest SES category corresponded to an approximate difference of 0.6–0.8 points on the LQ0–28 scale. Given the high mean QoL scores in this sample (approximately 22–24), this suggests a potential ceiling effect, limiting the ability to detect larger SES-related differences.

In this cohort, SES appears to be an important, but relatively small, contributor to QoL, with uncertain clinical relevance. Importantly, even modest SES-related differences may contribute to the observed group differences, supporting the likelihood of residual confounding at the school level.

### Waist-to-Height Ratio

WHtR was negatively associated with QoL, corresponding to an approximate reduction of 0.32 QoL points per 0.1 increase in WHtR. WHtR was also associated with lower levels of MVPA. These findings should be interpreted in light of previous research identifying WHtR as an important indicator of adverse health outcomes, including metabolic syndrome and cardiovascular risk factors [43].

Compared to MVPA, the magnitude of the association between WHtR and QoL was considerably larger, suggesting that anthropometric factors may have a stronger association with QoL than physical activity levels alone. The findings also indicate that WHtR may reflect underlying health-related or psychosocial factors relevant to QoL. For example, children with higher WHtR may experience social stigmatization or lower self-esteem related to body image, which may be associated with lower QoL [33].

### Limitations

The QoL scores in this study were generally high, with both children and parents reporting high levels of well-being, indicating a potential ceiling effect that may have limited the ability to detect meaningful changes or stronger associations over time. Although some differences were statistically significant, their clinical relevance appears limited given the high baseline levels.

A key limitation is the non-randomized allocation of intervention and control schools, which were located in different geographical areas with differing socioeconomic profiles. As SES is consistently associated with QoL, these baseline differences may have confounded the observed group differences. Although SES was included as a covariate in the regression analyses, this may not fully capture contextual differences at the school level. Residual confounding at the school or community level cannot therefore be ruled out. In addition, we did not include school-level modelling (e.g., random effects), which further limits the ability to fully account for contextual differences between schools. The differences in QoL between groups should therefore be interpreted with caution and not as causal effects of the intervention.

The additional 45 minutes of daily PA was associated with only small differences in QoL. This may reflect a combination of factors, including a modest association between PA and QoL in this age group, insufficient intensity or engagement in the intervention, participant attrition over time and a ceiling effect limiting detectable change.

SES was operationalised using fathers’ education, which may not fully capture the socioeconomic context of the household. Sensitivity analyses using alternative indicators, including maternal education and highest parental education, yielded similar results, supporting the robustness of the findings across SES specifications.

Although some overall terms in the child-reported models were not estimable, these analyses were retained because child-reported outcomes are central to the study question. The non-estimability appeared to be due to sparse data in specific group-by-year combinations, particularly for the intervention group in 2016. The models still yielded interpretable estimates for several continuous covariates, including MVPA and test year, although inference for overall group and interaction terms should be interpreted with caution.

Finally, reliance on parental proxy reports and changes in participant composition over time may have introduced bias and affected the stability of the estimates.

## Conclusion

This study examined the associations between a 5-year school-based physical activity intervention and quality of life in children aged 6–12 years. The findings indicated that age, socioeconomic status, and waist-to-height ratio were significantly associated with quality of life, while sex was not a significant predictor. Quality of life scores were generally high and remained stable or showed slight increases over time across both control and intervention groups, in contrast to previous research suggesting a decline in quality of life during childhood and adolescence.

Control schools, located in higher socioeconomic areas, had higher quality of life scores than intervention schools, highlighting the importance of contextual and socioeconomic differences. These differences should be interpreted with caution given underlying variations in socioeconomic context. Moderate-to-vigorous physical activity, waist-to-height ratio, and socioeconomic status were all significantly associated with quality of life, reflecting the multifactorial nature of children’s well-being.

Overall, the findings suggest that school-based physical activity initiatives may contribute to children’s well-being, although these associations appear modest and are influenced by contextual and socioeconomic factors.

## Abbreviations

HOPP: The Health Oriented Pedagogical Project
ILC: Inventory of Life Quality in Children and Adolescents
LQ0-28: Score for the Inventory of Life Quality in Children and Adolescents
PA: Physical activity
MVPA: Moderate-to-vigorous physical activity
QoL: Quality of life
SES: Father’s education level
SPSS: Statistical Package for Social Science
WHtR: Waist-to-height ratio

## References

[1] Ben-Arieh A, Casas F, Frønes I, Korbin J. Multifaceted concept of child well- being. Handbook of child well- being: Theories, methods and policies in global perspective. Dortmund: Springer Verlag. 2014:1–27. doi: 10.1007/978-90-481-9063-8_134

[2] Wallander JL, Koot HM. Quality of life in children: A critical examination of concepts, approaches, issues, and future directions. Clin Psychol Rev. 2016;45:131–43. doi: 10.1016/j.cpr.2015.11.007 26911191

[3] The World Health Organization Quality of Life (WHOQOL). https://www.who.int/publications/i/item/WHO-HIS-HSI-Rev.2012.03 2012. Accessed 2024 July 4.

[4] Ventegodt S, Andersen NJ, Merrick J. Quality of life philosophy I. Quality of life, happiness, and meaning in life. ScientificWorldJournal. 2003;3:1164–75. doi: 10.1100/tsw.2003.102 14646011 PMC5974893

[5] Del Pozo-Cruz B, Perales F, Parker P, Lonsdale C, Noetel M, Hesketh KD. Joint physical-activity/screen-time trajectories during early childhood: socio-demographic predictors and consequences on health-related quality-of-life and socio-emotional outcomes. Int J Behav Nutr Phys Act. 2019;16(1):55. 31286983 10.1186/s12966-019-0816-3 PMC6615223

[6] Winter K, Moor I, Markert J, Bilz L, Bucksch J, Dadaczynski K, et al. Concept and methodology of the Health Behaviour in School-aged Children (HBSC) study - Insights into the current 2022 survey and trends in Germany. J Health Monit. 2024;9(1):99–117. 38559683 10.25646/11878 PMC10977469

[7] Ringdal K, Ringdal GI, Olsen HK, Mamen A, Fredriksen PM. Quality of life in primary school children: The Health Oriented Pedagogical Project (HOPP). Scand J Public Health. 2018;46(21_suppl):68–73. doi: 10.1177/1403494818767821 29754578

[8] Land KC, Michalos AC, Sirgy MJ. Handbook of Social Indicators and Quality of Life Research. Springer Science & Business Media. 2011.

[9] Bakken A. Ungdata 2020: Nasjonale resultater. OsloMet. 2020. https://oda.oslomet.no/oda-xmlui/handle/20.500.12199/6415

[10] Løvgren M, Svagård V. Ungdata junior 2017–2018. Resultater fra en spørreundersøkelse blant elever i alderen 10 til 12 år. OsloMet. 2019. https://oda.oslomet.no/oda-xmlui/handle/20.500.12199/1327

[11] Casas F, Castella Sarriera J, Alfaro J, Gonzalez M, Malo S, Bertran I. Testing the personal wellbeing index on 12-16 year-old adolescents in 3 countries with 2 new items. Social Indicators Research. 2012;105:461–82.

[12] Michel G, Bisegger C, Fuhr DC, Abel T, KIDSCREEN group. Age and gender differences in health-related quality of life of children and adolescents in Europe: a multilevel analysis. Qual Life Res. 2009;18(9):1147–57. doi: 10.1007/s11136-009-9538-3 19774493

[13] Samdal O, Høivik Bye H, Torsheim T, Birkeland M, Diseth A, Fismen A, et al. Social inequality in health and learning among children and adolescents (Sosial ulikhet i helse og læring blant barn og unge). HEMIL-senteret Bergen: Universitetet i Bergen; 2012:272. Report No.: HEMIL-rapport 2/2012. Available from: http://hdl.handle.net/1956/6809

[14] da Costa B, da Costa RM, de Mello GT, Bandeira AS, Chaput J-P, Silva KS. Independent and joint associations of cardiorespiratory fitness and weight status with health-related quality of life among Brazilian adolescents. Qual Life Res. 2023;32(7):2089–98. doi: 10.1007/s11136-023-03379-0 36856892

[15] Albrecht GL, Devlieger PJ. The disability paradox: high quality of life against all odds. Soc Sci Med. 1999;48(8):977–88. doi: 10.1016/s0277-9536(98)00411-0 10390038

[16] Michalos AC. Connecting the quality of life theory to health, well-being and education. Cham: Springer International Publishing. 2017.

[17] Solans M, Pane S, Estrada M-D, Serra-Sutton V, Berra S, Herdman M, et al. Health-related quality of life measurement in children and adolescents: a systematic review of generic and disease-specific instruments. Value Health. 2008;11(4):742–64. doi: 10.1111/j.1524-4733.2007.00293.x 18179668

[18] Jozefiak T, Larsson B, Wichstrøm L, Mattejat F, Ravens-Sieberer U. Quality of Life as reported by school children and their parents: a cross-sectional survey. Health Qual Life Outcomes. 2008;6:34. doi: 10.1186/1477-7525-6-34 18489777 PMC2409303

[19] Mattejat F, Remschmidt H. ILK - Das Inventar zur Erfassung der Lebensqualität bei Kindern und Jugendlichen (ILK) - (The inventory of life quality in children and adolescents ILC). Bern: Hans Huber Verlag. 2006. 9757529

[20] Fredriksen PM, Olsen HK, Johansen Meza T. Changes in quality of life in elementary school children - the health oriented pedagogical project (HOPP). Sports. 2019;7(1). 10.3390/sports7010011 PMC6360024 30609845

[21] Jozefiak T, Larsson B, Wichstrøm L, Rimehaug T. Competence and emotional/behavioural problems in 7-16-year-old Norwegian school children as reported by parents. Nord J Psychiatry. 2012;66(5):311–9. doi: 10.3109/08039488.2011.638934 22171934

[22] Kristensen H, Hove P. Måleegenskaper ved den norske versjonen av Inventory of Life Quality in Children and Adolescents. PsykTestBarn. 2013;1(5):1–8.

[23] Marker AM, Steele RG, Noser AE. Physical activity and health-related quality of life in children and adolescents: A systematic review and meta-analysis. Health Psychol. 2018;37(10):893–903. doi: 10.1037/hea0000653 30234348

[24] Bull FC, Al-Ansari SS, Biddle S, Borodulin K, Buman MP, Cardon G, et al. World Health Organization 2020 guidelines on physical activity and sedentary behaviour. Br J Sports Med. 2020;54(24):1451–62. doi: 10.1136/bjsports-2020-102955 33239350 PMC7719906

[25] Hallal PC, Martínez-Mesa J, Coll CV, Mielke GI, Mendes MA, Peixoto MB, et al. Physical Activity at 11 Years of Age and Incidence of Mental Health Problems in Adolescence: Prospective Study. J Phys Act Health. 2015;12(4):535–9. doi: 10.1123/jpah.2013-0029 25347914

[26] Howie EK, McVeigh JA, Smith AJ, Zabatiero J, Bucks RS, Mori TA, et al. Physical activity trajectories from childhood to late adolescence and their implications for health in young adulthood. Prev Med. 2020;139:106224. doi: 10.1016/j.ypmed.2020.106224 32735989

[27] Rodriguez-Ayllon M, Cadenas-Sánchez C, Estévez-López F, Muñoz NE, Mora-Gonzalez J, Migueles JH, et al. Role of Physical Activity and Sedentary Behavior in the Mental Health of Preschoolers, Children and Adolescents: A Systematic Review and Meta-Analysis. Sports Med. 2019;49(9):1383–410. doi: 10.1007/s40279-019-01099-5 30993594

[28] Opdal IM, Morseth B, Handegård BH, Lillevoll K, Ask H, Nielsen CS, et al. Change in physical activity is not associated with change in mental distress among adolescents: the Tromsø study: Fit Futures. BMC Public Health. 2019;19(1):916. doi: 10.1186/s12889-019-7271-6 31288796 PMC6617649

[29] Van Dijk ML, Savelberg HHCM, Verboon P, Kirschner PA, De Groot RHM. Decline in physical activity during adolescence is not associated with changes in mental health. BMC Public Health. 2016;16:300. 27056368 10.1186/s12889-016-2983-3 PMC4825085

[30] Straatmann VS, Oliveira AJ, Rostila M, Lopes CS. Changes in physical activity and screen time related to psychological well-being in early adolescence: findings from longitudinal study ELANA. BMC Public Health. 2016;16:977. doi: 10.1186/s12889-016-3606-8 27630121 PMC5024465

[31] Khan A, Lee E-Y, Tremblay MS. Meeting 24-h movement guidelines and associations with health related quality of life of Australian adolescents. J Sci Med Sport. 2021;24(5):468–73. doi: 10.1016/j.jsams.2020.10.017 33229218

[32] Omorou AY, Langlois J, Lecomte E, Briançon S, Vuillemin A. Cumulative and bidirectional association of physical activity and sedentary behaviour with health-related quality of life in adolescents. Qual Life Res. 2016;25(5):1169–78. doi: 10.1007/s11136-015-1172-7 26542533

[33] Robinson TN, Chang JY, Haydel KF, Killen JD. Overweight concerns and body dissatisfaction among third-grade children: the impacts of ethnicity and socioeconomic status. J Pediatr. 2001;138(2):181–7. doi: 10.1067/mpd.2001.110526 11174614

[34] Eddolls WTB, McNarry MA, Lester L, Winn CON, Stratton G, Mackintosh KA. The association between physical activity, fitness and body mass index on mental well-being and quality of life in adolescents. Qual Life Res. 2018;27(9):2313–20. doi: 10.1007/s11136-018-1915-3 29948603 PMC6132966

[35] Dalene KE, Anderssen SA, Ekelund U, Thorén A-KH, Hansen BH, Kolle E. Permanent play facility provision is associated with children’s time spent sedentary and in light physical activity during school hours: A cross-sectional study. Prev Med Rep. 2016;4:429–34. doi: 10.1016/j.pmedr.2016.08.011 27583201 PMC4995570

[36] Deng WH, Fredriksen PM. Objectively assessed moderate-to-vigorous physical activity levels among primary school children in Norway: The Health Oriented Pedagogical Project (HOPP). Scand J Public Health. 2018;46(21_suppl):38–47. doi: 10.1177/1403494818771207 29754576

[37] Fredriksen PM, Hjelle OP, Mamen A, Meza TJ, Westerberg AC. The health Oriented pedagogical project (HOPP) - a controlled longitudinal school-based physical activity intervention program. BMC Public Health. 2017;17(1):370. doi: 10.1186/s12889-017-4282-z 28454531 PMC5410047

[38] Kharlova I, Fredriksen MV, Mamen A, Fredriksen PM. Daily and Weekly Variation in Children’s Physical Activity in Norway: A Cross-Sectional Study of The Health Oriented Pedagogical Project (HOPP). Sports (Basel). 2020;8(11):150. doi: 10.3390/sports8110150 33233516 PMC7699509

[39] Slootmaker SM, Schuit AJ, Chinapaw MJ, Seidell JC, van Mechelen W. Disagreement in physical activity assessed by accelerometer and self-report in subgroups of age, gender, education and weight status. Int J Behav Nutr Phys Act. 2009;6:17. 19320985 10.1186/1479-5868-6-17 PMC2670257

[40] Troiano RP, Berrigan D, Dodd KW, Mâsse LC, Tilert T, McDowell M. Physical activity in the United States measured by accelerometer. Med Sci Sports Exerc. 2008;40(1):181–8. doi: 10.1249/mss.0b013e31815a51b3 18091006

[41] Mattejat F, Remschmidt H, Jozefiak T. ILC - Inventory of Life Quality in Children and Adolescents. Hogrefe. 2006.

[42] Hogrefe. ILC Inventory of Life Quality in Children and Adolescents. https://hogrefe.no/produkt/inventory-of-life-quality-in-children-and-adolescents-ilc/ 2025. Accessed 2025 January 17.

[43] Fredriksen PM, Skår A, Mamen A. Waist circumference in 6-12-year-old children: The Health Oriented Pedagogical Project (HOPP). Scand J Public Health. 2018;46(21_suppl):12–20. doi: 10.1177/1403494818767790 29754573

