# Supplementary material for: Physical activity and quality of life in children: Findings from the Health Oriented Pedagogical Project (HOPP)
Source: PLoS One. 2026 Jul 10;21(7):e0353686. doi: 10.1371/journal.pone.0353686 (PMC13354104; doi:10.1371/journal.pone.0353686)
Supplement: Attachment — Submitted filename: PONE-D-25-65082.pdf [file pone.0353686.s001.pdf]

PLOS One

The impact of physical activity on the quality of life in children – the Health Oriented Pedagogical Project (HOPP)

--Manuscript Draft--

**Manuscript Number:**	PONE-D-25-65082
**Article Type:**	Research Article
**Full Title:**	The impact of physical activity on the quality of life in children – the Health Oriented Pedagogical Project (HOPP)
**Short Title:**	Physical activity and QoL in children (HOPP)
**Corresponding Author:**	Rein Magnus Jensen, M.D.<br>Oslo Metropolitan University: OsloMet - storbyuniversitetet<br>Oslo, NORWAY
**Keywords:**	quality of life, physical activity, school, children
**Abstract:**	Purpose: The purpose of this study was to evaluate the effect of a 5-year school-based physical activity (PA) intervention on the quality of life (QoL) in children, aged 6-12 years old.<br>Methods: Data were collected through the Health Oriented Pedagogical Project (HOPP), a longitudinal study conducted in Norway. HOPP involved children and their parents from nine elementary schools (n=2,140 children and 1,639 parents completed the QoL-questionnaire). QoL was measured using the Inventory of Life Quality in Children and Adolescents (ILC), and physical activity (PA) was assessed using accelerometers. Additionally, parental education level (SES), waist-to-height ratio (WHtR), were also included.<br>Results: The analysis revealed a significant positive effect of PA on QoL ($\beta = 0.008$, $p < 0.001$), although the effect size was small. SES significantly influenced QoL in intervention schools ($\beta = 0.249$, $p < 0.001$), while WHtR negatively affected QoL in both groups, with a stronger impact in control schools ($\beta = -4.344$, $p = 0.010$). The control schools exhibited higher QoL scores than the intervention schools, with an average 0.5-point advantage ($\beta = 0.458$, $p < 0.001$), likely due to SES differences.<br>Conclusion: This study highlights the complex interplay between PA, SES, WHtR, and QoL in children. While PA is associated with better QoL, its impact is influenced by factors such as age, SES, and WHtR, with no significant effect observed for sex. The findings underscore the importance of sustained and varied physical engagement in school settings to enhance QoL. Moreover, interventions should account for multiple individual and environmental factors, particularly SES and physical health metrics, to optimize outcomes for diverse populations.
**Order of Authors:**	Rein Magnus Jensen, M.D.
	Per Morten Fredriksen, Professor
	Asgeir Mamen, Professor
	Christoffer Wang, M.D.
**Additional Information:**	
**Question**	**Response**
**Financial Disclosure**<br><br>Enter a financial disclosure statement that describes the sources of funding for the work included in this submission. Review the submission guidelines for detailed requirements. View published research articles from *PLOS ONE* for specific examples.	Yes

Powered by Editorial Manager® and ProduXion Manager® from Aries Systems Corporation

This statement is required for submission and **will appear in the published article** if the submission is accepted. Please make sure it is accurate.<br><br>**Funded studies**<br>Enter a statement with the following details:<br>• Initials of the authors who received each award<br>• Grant numbers awarded to each author<br>• The full name of each funder<br>• URL of each funder website<br>• Did the sponsors or funders play any role in the study design, data collection and analysis, decision to publish, or preparation of the manuscript?<br><br>Did you receive funding for this work?	
Please add funding details.<br>as follow-up to "**Financial Disclosure**<br><br>Enter a financial disclosure statement that describes the sources of funding for the work included in this submission. Review the submission guidelines for detailed requirements. View published research articles from *PLOS ONE* for specific examples.<br><br>This statement is required for submission and **will appear in the published article** if the submission is accepted. Please make sure it is accurate.<br><br>**Funded studies**<br>Enter a statement with the following details:<br>• Initials of the authors who received each award<br>• Grant numbers awarded to each author<br>• The full name of each funder<br>• URL of each funder website<br>• Did the sponsors or funders play any role in the study design, data collection and analysis, decision to publish, or preparation of the manuscript?	The HOPP research project was funded by Horten municipality, Kristiania University College, The Norwegian Order of Odd Fellow Research Fund, The Oslofjord Regional Research Fund, and the Norwegian Fund for Post-Graduate Training in Physiotherapy.

Did you receive funding for this work?"	
Please select the country of your main research funder (please select carefully as in some cases this is used in fee calculation).<br>as follow-up to "**Financial Disclosure**<br><br>Enter a financial disclosure statement that describes the sources of funding for the work included in this submission. Review the submission guidelines for detailed requirements. View published research articles from *PLOS ONE* for specific examples.<br><br>This statement is required for submission and **will appear in the published article** if the submission is accepted. Please make sure it is accurate.<br><br>**Funded studies**<br>Enter a statement with the following details:<br>• Initials of the authors who received each award<br>• Grant numbers awarded to each author<br>• The full name of each funder<br>• URL of each funder website<br>• Did the sponsors or funders play any role in the study design, data collection and analysis, decision to publish, or preparation of the manuscript?<br><br>Did you receive funding for this work?"	NORWAY - NO
**Competing Interests**<br><br>Use the instructions below to enter a competing interest statement for this submission. On behalf of all authors, disclose any competing interests that could be perceived to bias this work—acknowledging all financial support and any other relevant financial or non-financial competing interests.<br><br>This statement is required for submission	The authors have declared that no competing interests exist.

and **will appear in the published article** if the submission is accepted. Please make sure it is accurate and that any funding sources listed in your Funding Information later in the submission form are also declared in your Financial Disclosure statement.<br><br>View published research articles from *PLOS ONE* for specific examples.<br><br>**NO authors have competing interests**<br><br>Enter: *The authors have declared that no competing interests exist.*<br><br>**Authors with competing interests**<br><br>Enter competing interest details beginning with this statement:<br><br>*I have read the journal's policy and the authors of this manuscript have the following competing interests: [insert competing interests here]*<br><br>* typeset	
**Ethics Statement**<br><br>Enter an ethics statement for this submission. This statement is required if the study involved:<br><br>• Human participants<br>• Human specimens or tissue<br>• Vertebrate animals or cephalopods<br>• Vertebrate embryos or tissues<br>• Field research<br><br>Write "N/A" if the submission does not require an ethics statement.<br><br>General guidance is provided below. Consult the submission guidelines for detailed instructions. **Make sure that all information entered here is included in the Methods section of the manuscript.**	The Regional Committees for Medical and Health Research Ethics approved the study protocol (reference number: 2014/2064/REK sør-øst). The approval date was January 9, 2015. Written informed consent from the participants and their parents or caretakers was obtained prior to data collection.

Powered by Editorial Manager® and ProduXion Manager® from Aries Systems Corporation

**Format for specific study types**<br><br>**Human Subject Research (involving human participants and/or tissue)**<br>• Give the name of the institutional review board or ethics committee that approved the study<br>• Include the approval number and/or a statement indicating approval of this research<br>• Indicate the form of consent obtained (written/oral) or the reason that consent was not obtained (e.g. the data were analyzed anonymously)<br><br>**Animal Research (involving vertebrate animals, embryos or tissues)**<br>• Provide the name of the Institutional Animal Care and Use Committee (IACUC) or other relevant ethics board that reviewed the study protocol, and indicate whether they approved this research or granted a formal waiver of ethical approval<br>• Include an approval number if one was obtained<br>• If the study involved *non-human primates*, add *additional details* about animal welfare and steps taken to ameliorate suffering<br>• If anesthesia, euthanasia, or any kind of animal sacrifice is part of the study, include briefly which substances and/or methods were applied<br><br>**Field Research**<br><br>Include the following details if this study involves the collection of plant, animal, or other materials from a natural setting:<br>• Field permit number<br>• Name of the institution or relevant body that granted permission	
**Data Availability**<br><br>Authors are required to make all data underlying the findings described fully available, without restriction, and from the time of publication. PLOS allows rare exceptions to address legal and ethical concerns. See the PLOS Data Policy and FAQ for detailed information.	Yes - all data are fully available without restriction

Powered by Editorial Manager® and ProduXion Manager® from Aries Systems Corporation

A Data Availability Statement describing where the data can be found is required at submission. Your answers to this question constitute the Data Availability Statement and **will be published in the article**, if accepted.<br><br>**Important:** Stating 'data available on request from the author' is not sufficient. If your data are only available upon request, select 'No' for the first question and explain your exceptional situation in the text box.<br><br>Do the authors confirm that all data underlying the findings described in their manuscript are fully available without restriction?	
**Describe where the data may be found in full sentences. If you are copying our sample text, replace any instances of XXX with the appropriate details.**<br><br>• If the data are **held or will be held in a public repository**, include URLs, accession numbers or DOIs. If this information will only be available after acceptance, indicate this by ticking the box below. For example: *All XXX files are available from the XXX database (accession number(s) XXX, XXX.*).<br>• If the data are all contained **within the manuscript and/or Supporting Information files**, enter the following: *All relevant data are within the manuscript and its Supporting Information files.*<br>• If neither of these applies but you are able to provide **details of access elsewhere**, with or without limitations, please do so. For example:<br><br>*Data cannot be shared publicly because of [XXX]. Data are available from the XXX Institutional Data Access / Ethics Committee (contact via XXX) for researchers who meet the criteria for access to confidential data.*<br><br>*The data underlying the results presented in the study are available from (include the name of the third party*	Publicly available data: The datasets generated and/or analyzed during the current study are available in the Norwegian Directorate for Education and Training (https://www.regjeringen.no/en/dep/kd/organisation/kunnskapsdepartementets-etater-og-virksomheter/Subordinate-agencies-2/norwegian-directorate-for-education-and-/id426533/).

Powered by Editorial Manager® and ProduXion Manager® from Aries Systems Corporation

and contact information or URL).<br>• This text is appropriate if the data are owned by a third party and authors do not have permission to share the data.<br><br>* typeset	
Additional data availability information:	

Manuscript

Click here to access/download;Manuscript;Impact of physical activity on QoL PLOS ONE.docx

Title page

The impact of physical activity on the quality of life in children – the Health Oriented Pedagogical Project (HOPP)

Author information

Corresponding author: Rein Magnus Jensen (ORCID: 0009-0003-0030-7029), Faculty of Education and International Studies, Oslo Metropolitan University (OsloMet), Oslo, Norway. reijen@oslomet.no

Asgeir Mamen (ORCID: 0000-0002-4316-7455), Department of Health and exercise, School of Health Sciences, Kristiania University College, Oslo, Norway.

Christoffer Wang, Oslo Metropolitan University (OsloMet), Faculty of Education and International Studies, Oslo, Norway.

Per Morten Fredriksen (ORCID: 0000-0001-7450-2925), Faculty of Applied Ecology, Agricultural Sciences and Biotechnology, Inland Norway University of Applied Sciences, Hamar, Norway and Faculty of Health, Welfare, and organization, Østfold University College, Fredrikstad, Norway.

Trial registration

The study is registered in Clinical Trials (ClinicalTrials.gov Identifier: NCT02495714). as of June 20th – 2015, retrospectively registered. The collection of baseline values was initiated in mid-January 2015.

Keywords: quality of life, physical activity, school, children

The Impact of Physical Activity on the Quality of Life in Children – The Health Oriented Pedagogical Project (HOPP)

Abstract:

Purpose: The purpose of this study was to evaluate the effect of a 5-year school-based physical activity (PA) intervention on the quality of life (QoL) in children, aged 6-12 years old.

Methods: Data were collected through the Health Oriented Pedagogical Project (HOPP), a longitudinal study conducted in Norway. HOPP involved children and their parents from nine elementary schools (n=2,140 children and 1,639 parents completed the QoL-questionnaire). QoL was measured using the Inventory of Life Quality in Children and Adolescents (ILC), and physical activity (PA) was assessed using accelerometers. Additionally, parental education level (SES), waist-to-height ratio (WHtR), were also included.

Results: The analysis revealed a significant positive effect of PA on QoL ($\beta = 0.008$, $p < 0.001$), although the effect size was small. SES significantly influenced QoL in intervention schools ($\beta = 0.249$, $p < 0.001$), while WHtR negatively affected QoL in both groups, with a stronger impact in control schools ($\beta = -4.344$, $p = 0.010$). The control schools exhibited higher QoL scores than the intervention schools, with an average 0.5-point advantage ($\beta = 0.458$, $p < 0.001$), likely due to SES differences.

Conclusion: This study highlights the complex interplay between PA, SES, WHtR, and QoL in children. While PA is associated with better QoL, its impact is influenced by factors such as age, SES, and WHtR, with no significant effect observed for sex. The findings underscore the importance of sustained and varied physical engagement in school settings to enhance QoL. Moreover, interventions should account for multiple individual and environmental factors, particularly SES and physical health metrics, to optimize outcomes for diverse populations.

Keywords: quality of life, physical activity, school, children

Abbreviations:

HOPP: The Health Oriented Pedagogical Project

ILC: Inventory of Life Quality in Children and Adolescents

LQ0-28: Score for the Inventory of Life Quality in Children and Adolescents.

PA: Physical activity

QoL: Quality of life

SES: Fathers education level

SPSS: Statistical Package for Social Science

WHtR: Waist-to-height ratio

Introduction

Identifying and addressing negative societal trends is essential for initiating necessary interventions and implementing countermeasures. Shifts in a population's quality of life hold significant importance for various stakeholders, including health authorities, policymakers, school counselors, child and family therapists, and parents [1]. To understand what affects children's well-being over time, it is essential to conduct epidemiological studies within cohorts of healthy children [2].

The World Health Organization (WHO) defines Quality of Life (QoL) as an individual's perception of their position in life in the context of the culture and value systems in which they live and in relation to their goals, expectations, standards, and concerns [3]. QoL is a broad and multifaceted term encompassing various aspects of life, such as physical, social, and emotional dimensions [4]. QoL can be measured subjectively, on the basis of individual evaluations, or objectively, based on measurable factors such as physical fitness and private economy [2]. For example, studies indicate that children

from low-income families tend to have lower QoL scores compared to children from high-income families [5,6]. Furthermore, the baseline results from the Health Oriented Pedagogical Project (HOPP) revealed a strong connection between parents education level and better QoL [7] which aligns with similar research on this topic [8] and is what we use in this article as SES.

In Norway, reports on children aged 10-12 years by the Norwegian Social Research Institute (NOVA) from 2019 and 2020 on adolescents in secondary and high schools, show that approximately 90% of children and adolescents are satisfied with life [9,10]. Norwegian studies have shown a stable or slight decrease in satisfaction with life from childhood to adolescence, and in general, girls (15%) are less satisfied than boys (10%) [9,10]. International studies have also revealed a decrease in QoL with increasing age in adolescents [11–13].

QoL and ILC

Health is a central component of QoL and has consistently been associated with both physical health and mental health outcomes [14] and is therefore considered a separate measure of perceived life satisfaction, termed health-related quality of life (HRQoL) [15,16]. The introduction of HRQoL marked a shift from looking at health and well-being as the absence of illness to a broader perspective that considers factors that provide value and meaning [2].

In recent decades interest in developing multidimensional QoL instruments for children and adolescents that cover not only physical functioning, but also mental health has increased [17]. Both are assessed in the standardized Inventory of Life Quality in Children and Adolescents (ILC) [18,19]. The ILC inventory was originally developed for children with mental or physical health problems, but it has since been adapted to the general population, and may be used to investigate results across decades [20]. The ILC inventory addresses the child’s subjective experience of their life situation, with particular emphasis on health condition, general functioning, social integration, and participation in age-specific activities [21]. Specifically, ILC assesses perceived QoL in general, and six items address

physical and mental health, family and school functioning, social contact with peers, interests and recreational activities. ILC also comes with a corresponding parental version [22].

QoL, physical activity and health

The association between physical activity (PA) and quality of life (QoL) among children and adolescents is well-documented. PA has been shown to reduce mental health issues such as depression and anxiety while promoting prosocial behavior [24–26]. A comprehensive meta-analysis reported a small but significant effect size for PA interventions on QoL, with Cohen's $d = 0.173$ (95% CI: 0.106–0.239, $p < 0.001$). This effect was stronger among adolescents, whereas findings for younger children were less consistent [24–26].

An Australian study demonstrated that children maintaining high PA levels and low screen time achieved significantly higher QoL compared to those with low PA and high screen time [5]. Similarly, baseline data from the HOPP study highlighted that aerobic fitness positively influenced QoL [7].

Findings from the Tromsø study (Fit Futures) further supported the role of aerobic fitness in enhancing QoL. For every additional 300 meters covered in an endurance test, QoL increased by 3 points on the scale [23]. These results emphasize the central role of physical fitness in supporting well-being among children and adolescents. Research suggests that psychological well-being in early adolescence can predict PA levels two years later [30], and meeting PA recommendations of over 60 minutes of moderate-to-vigorous activity a day is associated with better self-reported QoL [31,32].

Issues concerning body mass, are not limited to adults or adolescents. A Californian study found that concerns about weight and body dissatisfaction are highly prevalent among third-grade girls and boys, across ethnicity and socioeconomic factors [33]. Research has also shown that overweight or obese individuals typically experience a poorer QoL. A study involving 576 adolescents found that vigorous PA, cardiorespiratory fitness, and BMI were related to higher scores on QoL while BMI

influenced QoL indirectly through its effects on depressive symptoms and cardiorespiratory fitness [34]. The baseline data from the HOPP-study, concluded that BMI and waist circumference were negatively associated with QoL [7].

Research on PA levels among Norwegian children and adolescents shows that 74% of children aged 10-12 years engage in sports as a leisure activity, with a slight male predominance [9]. Among adolescents, 79% report exercising more than once a week [10]. Research indicates age-related variations in PA, revealing a decline from early school years to secondary school, and further into high school [10,35,36].

The aim of this study was to evaluate the effect of a 5-year school-based PA intervention on QoL in children aged 6–12 years

Methods

Study Design

The data for the present study were collected as part of the larger HOPP-study, a longitudinal case-control intervention designed to examine the impact of increased PA within a broader pedagogical framework focusing on both health and academic outcomes [37]. The primary aim was to integrate increased PA into the educational curriculum, targeting children from 1st to 6th grade in elementary schools in Norway, particularly in the Horten municipality and the Oslo metropolitan area.

The intervention was inclusive, cost-effective, and designed to reach children across all socioeconomic backgrounds by utilizing existing school facilities and teacher resources. The intervention emphasized mastery and confidence by transforming passive, desk-based learning into active, movement-based activities designed to enhance both academic skills and health. The program added 225 minutes of moderate-to-high intensity PA weekly, replacing traditional desk-based learning with curriculum-aligned activities in language, mathematics, and English. These activities, tailored for

each grade (1–7), were organized into grade-specific "activity boxes," allowing teachers to seamlessly integrate PA into daily learning. Combined with the standard 90 minutes of physical education per week, the intervention provided a total of 315 minutes of weekly PA. Teachers reported daily on the duration and intensity of activities at the class level, but individual participation was not monitored. Control schools followed the standard curriculum, maintaining 90 minutes of physical education per week without additional PA initiatives.

Participation in the study required written parental consent, and children could withdraw at any time without providing an explanation. Data collection commenced in spring 2015 and continued until participants completed primary school in 2020. A detailed study protocol, including the library of activities, is available [37].

Population

Parents and children aged 6-12 years, from nine elementary schools were invited to participate. Seven primary schools in Horten municipality were designated as intervention schools, while the two control schools were located near the Oslo metropolitan area. Both regions are predominantly upper-middle-class, but the control schools are located in areas with comparatively higher socioeconomic status. In 2015, informed consent was obtained from parents of 2,297 children, representing 82% of the target population of 2,816. Among these participants, 2,140 children (93%) and 1,639 parents (71%) completed the QoL-questionnaire.

Data collection

Measurements of PA have often been questionnaire-based and retrospective in nature [27], which may be vulnerable to recall bias [38]. While questionnaire-based measures that have undergone rigorous validation are considered valid, sensitive, and reliable, they are not without limitations. Similarly, wearable devices such as accelerometers, despite being valuable tools for objective measurement, also come with their own set of limitations and biases, such as over- or under-

estimation of certain movement behaviors, particularly in young children, depending on the wear protocol and data processing [27,39]. The use of a combination questionnaires and wearables would be preferrable, however, in the present study accelerometers was the choice of method for studying PA in children.

Inventory of Life Quality in Children and Adolescents (ILC)

A Norwegian version of the 7-item Inventory of Life Circumstances (ILC) [21] was used to evaluate participants' quality of life over the past week. The ILC assesses a global quality of life (QoL) score and measures seven key life areas: school performance, family relations, peer relations, autonomy in play, physical health, mental health, and a global assessment of well-being.

Responses to each item are rated on a five-point Likert scale, ranging from 1 (no problem) to 5 (severe problem). The instrument generates scores for problematic life areas and overall quality of life, reflecting both the presence of problems (problem score) and a positive assessment of QoL across all seven areas [21].

Procedure

Parents were provided with a digitized version of the ILC questionnaire and informed consent through email or satchel post. Parents were asked to provide information about their educational level, age, gender, and relations to the child. The questionnaire was designed to ensure anonymity, and unique identification numbers were used for data collection.

For children, the questionnaire was completed during school hours, under supervision for younger pupils. Fourth to sixth-grade students were able to complete the form without assistance. ILC was completed anonymously using digital methods, with data securely transferred to the database during annual testing.

Physical activity

To assess PA, an activity monitor accelerometer (ActiGraph wGT3X-BT, ActiGraph LLC, Pensacola, FL, USA) was used. The device was attached to the right side of the child's hip with an elastic band for seven consecutive days, at all hours, unless injured, ill, showering, swimming, or absent from school. The sampling frequency was set to 100 Hz at 10 s epochs. A minimum of 8 hrs/day for one day of registered PA was required for data analysis. Nonwear time was excluded using the Troiano technique with 60 min of consecutive zeroes and a tolerance of 2 min of activity [40]. Valid hours were defined as 06:00–23:59 in ActiLife 6 (ActiGraph LLC, Pensacola, Florida, USA). Categorical division of PA levels was based on mean counts per minute (cpm) as sedentary (0–99 cpm), light (100–1999 cpm), moderate (2000–4999 cpm), and vigorous (≥ 5000 cpm), and the number of minutes in each intensity domain was recorded [40]. Moderate-to-vigorous physical activity (MVPA) was calculated by summing minutes in moderate and vigorous intensity domains divided by the number of valid days.

Ethical considerations

Informed consent was obtained from all parents before their inclusion. Approval for the study was granted by the Regional Committee for Medical and Health Research Unit (reference number 2014/2064/REK sør-øst), ensuring adherence to ethical standards in research. The study is registered as a clinical trial (ClinicalTrials.gov Identifier: NCT02495714). This analysis utilizes data from January 15th, 2015, to May 15th, 2020. The study was conducted in accordance with the Declaration of Helsinki.

Statistical analysis

ILC responses were aggregated into two scores based on the Norwegian manual provided by the ILC Nordic distributor, Hogrefe AB [41,42]. These scores are the problem score (PR0–7) and the QoL score (LQ0–100). The PR0–7 score is computed by dichotomizing responses to each of the seven items, where ratings of 1 or 2 are coded as 0 (no problems) and ratings of 3, 4, or 5 are coded as 1 (problem present). The LQ0–28 scale is derived from the PB35 raw score (the sum of all seven items rated on a

scale of 1 to 5), with higher LQ0–28 values indicating better quality of life. The raw total score ranges from 7 (minimum, all responses rated as 1) to 35 (maximum, all responses rated as 5). Subtracting the lowest possible raw score (7) from the highest (35) gives a range of 28, which forms the basis for the LQ0–28 scale. To calculate the QoL score (LQ0–100): Subtract the PR0–7 score from the maximum score of 7 to calculate the LQ0–7 score, reflecting the absence of problems. Convert the LQ0–7 score to a percentage by dividing it by 7 and multiplying by 100, yielding the LQ0–100 score. Higher LQ0–100 values indicate better quality of life. The number 24 may be used as a result in LQ0–28-score. To convert it to percentage, 24 is divided by 28 and multiplied with 100 giving 85.7 as a LQ0–100-score. A percentage of 85.7 indicate a high QoL.

The dataset includes baseline, secular and longitudinal trends in the same model. As the pupils from the 1st grade in 2015 have repeated the test in all years through to 2020, they have 5 years *longitudinal* follow-up. Similar for the 2nd year pupils in 2015, as they have 4 years longitudinal follow-up, and so forth for pupils at older age in 2015. At the same time the model includes all the data for each year, hence we also have *secular trends* with reduced number of pupils for each year of intervention.

ANOVA was used to test the effect of grade and intervention/control schools on problem score LQ0-28 for both children and parents on baseline values. Similarly, a linear regression backward model was used to estimate the effects of sex, MVPA, test year, SES, WHtR and control/intervention schools on the total QoL score (LQ0-28) used by ILC in children.

In Norway, socioeconomic status is commonly determined by parental education, with higher levels of education corresponding to higher SES. The educational attainment of parents serves as a reliable proxy for SES, reflecting both economic resources and social capital within households. In this study, SES was assessed using fathers' education level as a proxy indicator. According to data from Statistics Norway (SSB), SES is categorized based on educational levels: primary and secondary school (SES = 1), high school (SES = 2), bachelor's degree (SES = 3), and master's degree or higher (SES = 4).

All analyses were conducted with the Statistical Package for the Social Sciences (SPSS) version 28 (IBM, Armonk, NY, USA). An α-value of 0.05 was used for statistical significance.

Results

Baseline

Cross-sectional measurements in children using ANOVA at baseline show an increase in QoL with increasing age ($p<0.001$), as shown in Figure 1. However, the response from parents by proxy illustrates a nonsignificant change across age.

(Insert figure 1 here)

Figure 1. The baseline results of QoL for children (left) and parents by proxy (right) for QoL across age groups. Note the difference in scale as children scored themselves lower than parents by proxy.

In Table 1, the years of testing are displayed in the left column. The upper horizontal row reveals dual information: [1)] the cohort divided from 1st – 6th grade for the *baseline* values, and [2)] the number of years the pupils participated in the study (1-6 years) for *longitudinal* and *secular trends*. Baseline results are revealed along the 2015 horizontal upper row. Secular trends are displayed in the columns under each year in the study (1-6 years). The longitudinal results are presented along the diagonal axis from the 1st results in 2015 to the last result in 2020. The results are shown for the intervention and control schools separately.

Table 1. The results of Quality-of-Life data (LQ0-28) for the whole sample, divided into grades and intervention/control schools. The baseline data are listed horizontally along the year 2015. The longitudinal data are marked in light grey. The secular trends are listed at the bottom of each table. Cohen's d is given in the last row.

	**Grades**												
	**1**		**2**		**3**		**4**		**5**		**6**		
**Intervention**	**Mean**	**SD**	**Mean**	**SD**	**Mean**	**SD**	**Mean**	**SD**	**Mean**	**SD**	**Mean**	**SD**	
**2015**	22.76	3.39	22.77	3.24	22.96	3.14	22.45	5.44	23.57	2.67	23.62	2.99	**Baseline**
**2016**			22.20	4.10	22.88	3.75	23.16	3.75	23.13	3.08	23.76	2.81	
**2017**					22.91	3.28	23.33	3.37	23.50	2.61	23.52	3.04	
**2018**							23.02	3.58	23.46	3.25	23.47	2.81	
**2019**									23.17	2.98	23.22	2.87	
**2020**											23.18	3.09	**Longitudinal**
	22.76	3.39	22.49	3.67	22.92	3.39	22.99	4.04	23.37	2.92	23.46	2.94	**Secular**
**Control**													
**2015**	22.29	3.72	22.87	3.33	24.01	2.64	24.46	2.31	24.63	2.48	24.61	2.68	**Baseline**
**2016**			22.59	3.53	22.63	3.67	24.44	3.02	24.44	2.48	24.47	2.77	
**2017**					23.41	3.30	23.73	3.24	24.14	2.95	24.75	2.84	
**2018**							23.62	2.54	23.84	3.13	24.72	2.40	
**2019**									24.25	2.95	23.92	2.56	
**2020**											23.94	2.8	**Longitudinal**
	22.29	3.72	22.73	3.43	23.35	3.20	24.06	2.78	24.26	2.80	24.4	2.68	**Secular**
**Cohen's *d***													
**Intervention/ Control**	-0.04		-0.07		-0.13		-0.31		-0.31		-0.33		

To illustrate the difference between baseline, secular and longitudinal, the results are shown in three graphs (Figure 2), separated by intervention and control schools. The first graph of baseline measures uses grades 1-6 (6-11 years) on the X-axis. The longitudinal data display the number of years the children have been involved in the study on the X-axis. For secular trends the accumulation at each year of the study is displayed by 2015-2020 on the X-axis. To emphasize variations in the data, as the data is narrow relative to the absolute values, the Y-axis is superimposed.

(Insert figure 2 here)

Figure 2. The figures show the crude values of QoL (LQ0-28) for children from baseline (left), secular trends (middle) and longitudinal trends (right). The Y-axis is superimposed to better show the differences, and the x-axis, which is dependent, show either grade (baseline), number of years in the study (longitudinal) or the year the data were accumulated (secular).

Regression model

The linear regression model revealed a significant effect of control/intervention schools, MVPA, WHtR and parental education, and no effect of sex. The control schools had higher QoL scores than the intervention schools by almost half a point (β 0.458, p<0.001). Similarly, parental education (father) revealed that on a four-level educational scale of [1)] primary/secondary schools, [2)] high school, to [3)] bachelor's degree and [4)] master's degree or higher, the QoL increased by 0.2 points for each educational level (β 0.204, p<0.001). For MVPA, the effect was limited, but significant (β 0.006, p<0.001). The year of testing served as a variable for how many years the pupils were in the intervention. The year 2015 implies baseline values, and each year until 2020 indicates one additional year in intervention. Hence, 2020 implies five years in intervention. In the present model a significant effect was found for year in intervention, as 0.133 points higher (β 0.133, p<0.001) was shown for each year, indicating a total of 0.665 higher values across 5 years. The largest effect was shown by WHtR, illustrating a reduction in 3 QoL points for each ratio up (β -3.183, p<0.001) in WHtR.

Table 2. Results of a linear regression model using stepwise backward strategy*

Model	Variable	Beta	C. SE	C. beta	t	p-value
**1**	**Constant**	-247.99	62.336		-3.978	<0.001
	**Sex**	-0.32	0.090	-0.005	-0.351	0.725
	**Test year**	0.135	0.31	0.064	4.358	<0.001
	**WHtR**	-3.206	0.931	-0.050	-3.443	<0.001
	**AMVPA**	0.006	0.002	0.055	3.559	<0.001
	**SES**	0.204	0.055	0.057	3.697	<0.001
	**Control/intervention**	0.461	0.099	0.073	4.647	<0.001
**2**	**Constant**	-248.73	62.295		-3.993	<0.001
	**Test year**	0.135	0.031	0.064	4.372	<0.001
	**WHtR**	-3.183	0.929	-0.050	-3.427	<0.001
	**AMVPA**	0.006	0.002	0.057	3.754	<0.001
	**SES**	0.204	0.055	0.056	3.689	<0.001
	**Control/intervention**	0.458	0.099	0.073	4.634	<0.001

* Dependent variable: QoL (LQ0-28), waist-to-height ratio = WHtR, Average Moderate to Vigorous Physical Activity = AMVPA, Socioeconomic status = SES, Control school v Intervention school = Control/Intervention.

Intervention/control

Regression analysis indicated that higher parental education (β = 0.249, p<0.001) was associated with higher QoL among children in the intervention group. Similar findings, albeit less effect was displayed for MVPA (β 0.008, p<0.001) and years in intervention (β 0.136, p<0.001) with higher QoL with more PA and more years in intervention. WHtR (β -2.681, p=0.017), however, showed negative significant effects with lower QoL with higher WHtR. Sex (β -0.103, p=0.392) did not reach significance (Table 3). No effect was found for of parental education in the control schools (β 0.137, p=0.105). However, the number of years the children participated in the study (β 0.145, p=0.003) had a significant positive effect with improved QoL with older children. Higher WHtR (β -4.344, p=0.010) had a negative effect on QoL.

Table 3. Results of a linear regression model using stepwise backward strategy for intervention and control groups *

	Model	Variable	Beta	Coeff. SE	Coeff. beta	T	p-value
**Intervention**	**1**	**Constant**	-242.75	80.204		-3.027	0.002
		**Sex**	-0.103	0.120	-0.017	-0.857	0.392
		**Test year**	0.132	0.040	0.063	3.324	<0.001
		**WHtR**	-2.754	1.125	-0.046	-2.448	0.014
		**AMVPA**	0.008	0.002	0.069	3.478	<0.001
		**SES**	0.248	0.073	0.063	3.403	<0.001
	**2**	**Constant**	-246.67	80.070		-3.081	0.002
		**Test year**	0.134	0.040	0.064	3.375	<0.001
		**WHtR**	-2.681	1.122	-0.044	-2.389	0.017
		**AMVPA**	0.008	0.002	0.074	3.901	<0.001
		**SES**	0.249	0.073	0.063	3.423	<0.001
**Control**	**1**	**Constant**	-277.67	99.717		-2.783	0.005
		**Sex**	0.116	0.138	0.020	0.845	0.389
		**Test year**	0.150	0.049	0.071	3.035	0.002
		**WHtR**	-4.086	1.698	-0.056	-2.406	0.016
		**AMVPA**	0.003	0.002	0.031	1.303	0.193
		**SES**	0.128	0.085	0.035	1.512	0.131
	**2**	**Constant**	-276.62	99.703		-2.774	0.006
		**Test year**	0.150	0.049	0.071	3.029	0.002
		**WHtR**	-4.190	1.693	-0.057	-2.475	0.013
		**AMVPA**	0.003	0.002	0.027	1.179	0.239
		**SES**	0.136	0.084	0.037	1.609	0.108
	**3**	**Constant**	-259.81	98.689		-2.633	0.009
		**Test year**	0.142	0.049	0.067	2.891	0.004
		**WHtR**	-4.199	1.694	-0.057	-2.479	0.013
		**SES**	0.137	0.084	0.037	1.622	0.105
	**4**	**Constant**	-266.74	98.639		-2.704	0.007
		**Test year**	0.145	0.049	0.068	2.968	0.003
		**WHtR**	-4.344	1.692	-0.059	-2.567	0.010

*Depen[illegible] variable: QoL (LQ0-28), waist-to-height ratio = WHtR, Average Moderate to Vigorous Physical Activity = AMVPA, Socioeconomic status = SES, Control school v Intervention school = Control/Intervention.

Socioeconomic status (SES)

Using fathers' education as a proxy for SES it showed a positive significant role in children's QoL, as Figure 3 underlines. Both children and parents by proxy show a clear positive effect of SES on QoL for both sexes.

(Insert figure 3a and figure 3 b here)

Figure 3. The effect of SES on children's QoL. The children's response is shown on the left and parents by proxy on the right. Education level/SES equals primary/secondary school = 1, high school = 2, bachelor's degree = 3 and master's/PhD = 4.

Discussion

The purpose of this study was to evaluate the effect of a 5-year school-based PA intervention on the QoL in children, aged 6-12 years old. Our findings show that while PA, measured as MVPA, generally supports better QoL, its specific impact varies depending on age, sex, WHtR, and SES. This variation indicates that the contribution of PA to QoL is influenced by a combination of individual and environmental factors.

The results revealed a small but consistent improvement in QoL with age for both the intervention and control groups. This improvement contrasts with cross-sectional studies that have shown a decline in QoL with increasing age; alternatively, that QoL is relatively stable during childhood and adolescence [9–13].

New findings in light of baseline data

Our longitudinal data provide insights into the trajectory of QoL as influenced by PA. While baseline assessments identified aerobic fitness and muscular strength as significant contributors to QoL, our extended analysis suggests that the influence of PA on QoL, although statistically significant, is subtler than initially anticipated. This finding aligns with the baseline observation that not all physical attributes correlate directly with QoL improvements.

The findings indicate that the duration of participation in the intervention had a significant and cumulative positive effect on QoL. Each additional year in the intervention was associated with a increase in QoL culminating in a total increase of 0.7 points on the QoL-scale over a five-year period. This result underscores the importance of sustained engagement in school-based physical activity interventions, as prolonged exposure appears to amplify the benefits on QoL. Notably, the sex of the children did not significantly affect QoL scores, suggesting that the intervention provides equitable benefits across genders [9].

Regression model

The results from the linear regression backward model concerning children, indicate a significant influence of MVPA, WHtR, and SES on QoL. The education level of the children's parents, specifically the father, also had a significant effect on QoL. For each level increase on a four-level educational scale - from primary/secondary schools to high school, to bachelor's degree, to master's degree or higher - the QoL score increased significantly by 0.2 points. The children's level of MVPA had a limited, but still significant, effect on QoL. This is consistent with previous research, which has reported small effect sizes for the relationship between PA and QoL in children and adolescents [28, 29]. While PA is often positively associated with health-related quality of life (HRQoL), the magnitude of this relationship tends to be modest and varies across different populations [23]. Some studies have also found no significant changes in mental health outcomes with variations in PA levels among adolescents, suggesting that other factors, such as psychosocial support or school environments, may

play a more substantial role [29]. Worth mentioning is that the initial baseline study in the HOPP study posited a clear beneficial impact of physical fitness on QoL [7].

Our findings add to this body of work by indicating that the intensity and duration of PA may influence QoL more subtly than anticipated. Research has suggested that meeting recommended levels of MVPA, such as 60 minutes per day, may be necessary to observe significant benefits [31]. However, the small effect observed in this study highlights the importance of varied and sustained PA engagement to produce measurable improvements in QoL, as supported by earlier findings [27].

Intervention/control

Our analysis revealed differences between the intervention and control groups, which provides insights into the impact of the intervention and other influencing factors. In the intervention schools, key predictors of QoL included SES, MVPA, years in intervention and WHtR.

In control schools, SES did not significantly influence QoL, in contrast with the intervention schools, suggesting that SES impacts may have been mediated by our intervention. In contrast, years of study participation and WHtR were significant predictors of QoL in control schools. The consistent influence of WHtR across groups highlights the role of physical health metrics in QoL, emphasizing the importance of addressing these factors universally.

Notably, control schools exhibited higher QoL scores compared to intervention schools, with an average 0.5-point advantage. This result, though unexpected, highlights the importance of examining contextual factors that may have influenced these outcomes.

Sex did not emerge as a significant predictor, suggesting that the effects of the intervention were relatively similar across genders. This aligns with insights from previous reports from Norway [10], which showed limited gender differences in well-being among children aged 10–12 years. While gender disparities in psychosocial factors like self-esteem and stress begin to emerge during late childhood, these trends are more pronounced in adolescence [9, 10].

Socioeconomic status (SES)

The results suggest that while SES is a crucial factor in the intervention group, it does not play a similar role in the control groups. In control schools, QoL appears to be more strongly influenced by the duration of study participation, although no actual intervention, only the time involved, and the WHtR. As previously stated, the differences in outcomes between the control and intervention schools might be attributed to differences in SES of the two cohorts. Intervention schools were situated in a municipality known for lower SES, where smaller school sizes limited the representation of children from more advantaged backgrounds, despite two of the seven schools including parents with higher education. Conversely, the control schools were located closer to urban Oslo, benefiting from a parent population with higher education levels and thus enhanced SES. We propose that the demographic composition of control schools, potentially characterized by higher SES, better access to resources, and other community-level advantages, may have contributed to their higher QoL scores. These schools likely reflect a population already benefiting from supportive environments that promote enhanced QoL. These results are consistent with other findings that highlight the impact of socioeconomic status on health outcomes, as discussed in previous research [5].

Waist-to-Height Ratio

The WHtR had a substantial effect on QoL, as an increase in WHtR displayed there was a significant reduction in QoL of approximately three points. Similarly, WHtR, was indicative of lower MVPA levels. These results should be viewed in light of other studies that emphasize WHtR as a critical measure for assessing adverse health outcomes such as metabolic syndrome and cardiovascular risk factors [43]. Our analysis further proposes that WHtR may serve as a biological marker also for QoL. Perhaps children with higher WHtR face social stigmatization or suffer from lower self-esteem due to their body image, directly influencing their QoL [33].

Limitations

The results for QoL in this study are notably high, with both children and parents estimating the children's QoL to be excellent at the mean level. However, the variation between groups based on SES and test year is of limited clinical value, potentially due to a ceiling effect. At baseline, parental proxy reports indicated a slight decline in QoL over time, while child self-reports suggested an increase. This divergence may reflect increasing parental concerns as children grow older and become less influenced by parental oversight. Nevertheless, the changes observed were minimal, starting from high baseline values. These findings may still offer valuable reference points for comparison with individual results.

The introduction of 45 minutes of PA appeared to have little or no effect on perceived QoL in children, potentially due to several factors: 1) PA might not have the anticipated effect, 2) insufficient amounts of PA were implemented, 3) participant attrition resulted in unknown shifts in baseline characteristics as cohorts transitioned from primary to secondary school, 4) a ceiling effect limited the ability to detect changes, particularly evident in control schools, and 5) initial differences in SES may have biased results, as control schools were of higher SES.

The reliance on parental proxy reports for some measures may also have introduced bias, as parental perceptions of child well-being may not fully align with children's self-reports. Furthermore, fluctuations in participant numbers over time could have influenced the findings. Each year, one cohort left the study upon transferring to secondary school, which may have affected mean scores depending on whether lower- or higher-performing individuals exited the study.

Conclusion

This study evaluated the impact of a 5-year school-based PA intervention on the QoL of children aged 6–12 years. The findings revealed that age, SES, and WHtR were significant factors influencing the contribution of PA to QoL, while sex did not emerge as a significant predictor. A small but consistent improvement in QoL was observed with age across both control and intervention groups, which

contrasts with previous research suggesting a decline in QoL during childhood and adolescence. The high overall QoL estimates reported by both children and parents further highlight the robustness of these outcomes.

Control schools, located in higher SES areas, displayed higher QoL scores than intervention schools situated in a lower SES municipality. This result emphasizes the critical role of demographic and socioeconomic factors in shaping QoL. However, the observed increase in QoL in intervention schools can be considered a positive outcome, countering expectations of a decline in QoL from early school years to the end of elementary school. Factors such as MVPA, WHtR, and SES had significant but nuanced effects on QoL, highlighting the complexity of these relationships. These findings reinforce the importance of designing interventions that address multiple individual and environmental factors, particularly in lower SES contexts. Future research should explore how tailored strategies, and longer-term interventions can optimize QoL outcomes across diverse populations. These findings underscore the importance of considering multiple individual and environmental factors when designing interventions to enhance children's QoL.

Statements & Declarations

Ethics approval and consent to participate

The Regional Committees for Medical and Health Research Ethics approved the study protocol (reference number: 2014/2064/REK sør-øst). The approval date was January 9, 2015. Written informed consent from the participants and their parents or caretakers was obtained prior to data collection.

Consent for publication

Not applicable.

Availability of data and materials

Publicly available data: The datasets generated and/or analyzed during the current study are available in the Norwegian Directorate for Education and Training (https://www.regjeringen.no/en/dep/kd/organisation/kunnskapsdepartementets-etater-og-virksomheter/Subordinate-agencies-2/norwegian-directorate-for-education-and-/id426533/).

The datasets generated and/or analyzed during the current study are available from the corresponding author upon reasonable request.

Competing Interests

The authors declare that they have no competing interests.

Funding

The HOPP research project was funded by Horten municipality, Kristiania University College, The Norwegian Order of Odd Fellow Research Fund, The Oslofjord Regional Research Fund, and the Norwegian Fund for Post-Graduate Training in Physiotherapy. The funders had no role in study design, data collection and analysis, decision to publish, or preparation of the manuscript.

Author contributions

Conceptualization: All authors
Methodology: Per Morten Fredriksen, Asgeir Mamen
Data curation: Asgeir Mamen, Per Morten Fredriksen
Formal analysis: Asgeir Mamen
Investigation: Per Morten Fredriksen, Asgeir Mamen
Writing – original draft: Rein Magnus Jensen
Writing – review & editing: Rein Magnus Jensen, Christoffer Wang, Per Morten Fredriksen, Asgeir Mamen
Supervision: Per Morten Fredriksen

Acknowledgments
The authors thank the participating schools, teachers, students, and parents for their cooperation and commitment during data collection. The authors also acknowledge the support of the municipalities and school administrations involved in facilitating the implementation of the HOPP project.

Author information

Corresponding author: Rein Magnus Jensen (ORCID: 0009-0003-0030-7029), Faculty of Education and International Studies, Oslo Metropolitan University (OsloMet), Oslo, Norway. reijen@oslomet.no

Asgeir Mamen (ORCID: 0000-0002-4316-7455), Department of Health and exercise, School of Health Sciences, Kristiania University College, Oslo, Norway.

Christoffer Wang, Oslo Metropolitan University (OsloMet), Faculty of Education and International Studies, Oslo, Norway.

Per Morten Fredriksen (ORCID: 0000-0001-7450-2925), Faculty of Applied Ecology, Agricultural Sciences and Biotechnology, Inland Norway University of Applied Sciences, Hamar, Norway and Faculty of Health, Welfare, and organization, Østfold University College, Fredrikstad, Norway.

References

1. Ben-Arieh A, Casas F, Frønes I, Korbin J. Multifaceted concept of child well- being. In: Handbook of child well- being: Theories, methods and policies in global perspective [Internet]. Dortmund: Springer Verlag; 2014. p. 1–27. Available from: https://doi.org/10.1007/978-90-481-9063-8_134

2. Wallander JL, Koot HM. Quality of life in children: A critical examination of concepts, approaches, issues, and future directions. Clin Psychol Rev. 2016 Apr;45:131–43.

3. The World Health Organization Quality of Life (WHOQOL) [Internet]. 2012 [cited 2024 Jul 4]. Available from: https://www.who.int/publications/i/item/WHO-HIS-HSI-Rev.2012.03

4. Ventegodt S, Andersen NJ, Merrick J. Quality of life philosophy I. Quality of life, happiness, and meaning in life. ScientificWorldJournal. 2003 Dec 1;3:1164–75.

5. Del Pozo-Cruz B, Perales F, Parker P, Lonsdale C, Noetel M, Hesketh KD, et al. Joint physical-activity/screen-time trajectories during early childhood: socio-demographic predictors and consequences on health-related quality-of-life and socio-emotional outcomes. Int J Behav Nutr Phys Act. 2019 Jul 8;16(1):55.

6. Winter K, Moor I, Markert J, Bilz L, Bucksch J, Dadaczynski K, et al. Concept and methodology of the Health Behaviour in School-aged Children (HBSC) study - Insights into the current 2022 survey and trends in Germany. J Health Monit. 2024 Mar;9(1):99–117.

7. Ringdal K, Ringdal GI, Olsen HK, Mamen A, Fredriksen PM. Quality of life in primary school children: The Health Oriented Pedagogical Project (HOPP). Scand J Public Health. 2018 May;46(21_suppl):68–73.

8. Land KC, Michalos AC, Sirgy MJ. Handbook of Social Indicators and Quality of Life Research. Springer Science & Business Media; 2011. 594 p.

9. Bakken A. Ungdata 2020: Nasjonale resultater [Internet]. NOVA, OsloMet; 2020 [cited 2024 Jul 4]. Available from: https://oda.oslomet.no/oda-xmlui/handle/20.500.12199/6415

10. Løvgren M, Svagård V. Ungdata junior 2017–2018. Resultater fra en spørreundersøkelse blant elever i alderen 10 til 12 år. [Internet]. OsloMet; 2019 [cited 2024 Jul 4]. Available from: https://oda.oslomet.no/oda-xmlui/handle/20.500.12199/1327

11. Casas F, Castella Sarriera J, Alfaro J, Gonzalez M, Malo S, Bertran I, et al. Testing the personal wellbeing index on 12-16 year-old adolescents in 3 countries with 2 new items. Social Indicators Research. 2012;105:461–82.

12. Michel G, Bisegger C, Fuhr DC, Abel T, KIDSCREEN group. Age and gender differences in health-related quality of life of children and adolescents in Europe: a multilevel analysis. Qual Life Res. 2009 Nov;18(9):1147–57.

13. Samdal O, Høivik Bye H, Torsheim T, Birkeland M, Diseth A, Fismen A, et al. Social inequality in health and learning among children and adolescents (Sosial ulikhet i helse og læring blant barn og unge) [Internet]. HEMIL-senteret Bergen: Universitetet i Bergen; 2012 p. 272. Report No.: HEMIL-rapport 2/2012. Available from: http://hdl.handle.net/1956/6809

14. da Costa B, da Costa RM, de Mello GT, Bandeira AS, Chaput JP, Silva KS. Independent and joint associations of cardiorespiratory fitness and weight status with health-related quality of life among Brazilian adolescents. Qual Life Res. 2023 Jul;32(7):2089–98.

15. Albrecht GL, Devlieger PJ. The disability paradox: high quality of life against all odds. Soc Sci Med. 1999 Apr;48(8):977–88.

16. Michalos AC. Connecting the Quality of Life Theory to Health, Well-being and Education [Internet]. Cham: Springer International Publishing; 2017 [cited 2024 Jul 4]. Available from: http://link.springer.com/10.1007/978-3-319-51161-0

17. Solans M, Pane S, Estrada MD, Serra-Sutton V, Berra S, Herdman M, et al. Health-related quality of life measurement in children and adolescents: a systematic review of generic and disease-specific instruments. Value Health. 2008 Aug;11(4):742–64.

18. Jozefiak T, Larsson B, Wichstrøm L, Mattejat F, Ravens-Sieberer U. Quality of Life as reported by school children and their parents: a cross-sectional survey. Health Qual Life Outcomes. 2008;6:34.

19. Mattejat F, Remschmidt H. ILK - Das Inventar zur Erfassung der Lebensqualität bei Kindern und Jugendlichen (ILK) - (The inventory of life quality in children and adolescents ILC). Bern: Hans Huber Verlag; 2006.

20. Fredriksen PM, Olsen HK, Johansen Meza T. Changes in Quality of Life in Elementary School Children-The Health Oriented Pedagogical Project (HOPP). Sports (Basel). 2019 Jan 3;7(1).

21. Jozefiak T, Larsson B, Wichstrøm L, Rimehaug T. Competence and emotional/behavioural problems in 7-16-year-old Norwegian school children as reported by parents. Nord J Psychiatry. 2012 Oct;66(5):311–9.

22. Kristensen H, Hove P. Måleegenskaper ved den norske versjonen av Inventory of Life Quality in Children and Adolescents. PsykTestBarn. 2013 Apr;1(5):1–8.

23. Marker AM, Steele RG, Noser AE. Physical activity and health-related quality of life in children and adolescents: A systematic review and meta-analysis. Health Psychol. 2018 Oct;37(10):893–903.

24. Bull FC, Al-Ansari SS, Biddle S, Borodulin K, Buman MP, Cardon G, et al. World Health Organization 2020 guidelines on physical activity and sedentary behaviour. Br J Sports Med. 2020 Dec;54(24):1451–62.

25. Hallal PC, Martínez-Mesa J, Coll CV, Mielke GI, Mendes MA, Peixoto MB, et al. Physical Activity at 11 Years of Age and Incidence of Mental Health Problems in Adolescence: Prospective Study. J Phys Act Health. 2015 Apr;12(4):535–9.

26. Howie EK, McVeigh JA, Smith AJ, Zabatiero J, Bucks RS, Mori TA, et al. Physical activity trajectories from childhood to late adolescence and their implications for health in young adulthood. Prev Med. 2020 Oct;139:106224.

27. Rodriguez-Ayllon M, Cadenas-Sánchez C, Estévez-López F, Muñoz NE, Mora-Gonzalez J, Migueles JH, et al. Role of Physical Activity and Sedentary Behavior in the Mental Health of Preschoolers, Children and Adolescents: A Systematic Review and Meta-Analysis. Sports Med. 2019 Sep;49(9):1383–410.

28. Opdal IM, Morseth B, Handegård BH, Lillevoll K, Ask H, Nielsen CS, et al. Change in physical activity is not associated with change in mental distress among adolescents: the Tromsø study: Fit Futures. BMC Public Health. 2019 Jul 9;19(1):916.

29. Van Dijk ML, Savelberg HHCM, Verboon P, Kirschner PA, De Groot RHM. Decline in physical activity during adolescence is not associated with changes in mental health. BMC Public Health. 2016 Apr 7;16:300.

30. Straatmann VS, Oliveira AJ, Rostila M, Lopes CS. Changes in physical activity and screen time related to psychological well-being in early adolescence: findings from longitudinal study ELANA. BMC Public Health. 2016 Sep 15;16:977.

31. Khan A, Lee EY, Tremblay MS. Meeting 24-h movement guidelines and associations with health related quality of life of Australian adolescents. J Sci Med Sport. 2021 May;24(5):468–73.

32. Omorou AY, Langlois J, Lecomte E, Briançon S, Vuillemin A. Cumulative and bidirectional association of physical activity and sedentary behaviour with health-related quality of life in adolescents. Qual Life Res. 2016 May;25(5):1169–78.

33. Robinson TN, Chang JY, Haydel KF, Killen JD. Overweight concerns and body dissatisfaction among third-grade children: the impacts of ethnicity and socioeconomic status. J Pediatr. 2001 Feb;138(2):181–7.

34. Eddolls WTB, McNarry MA, Lester L, Winn CON, Stratton G, Mackintosh KA. The association between physical activity, fitness and body mass index on mental well-being and quality of life in adolescents. Qual Life Res. 2018 Sep;27(9):2313–20.

35. Dalene KE, Anderssen SA, Ekelund U, Thorén AKH, Hansen BH, Kolle E. Permanent play facility provision is associated with children's time spent sedentary and in light physical activity during school hours: A cross-sectional study. Prev Med Rep. 2016 Dec;4:429–34.

36. Deng WH, Fredriksen PM. Objectively assessed moderate-to-vigorous physical activity levels among primary school children in Norway: The Health Oriented Pedagogical Project (HOPP). Scand J Public Health. 2018 May;46(21_suppl):38–47.

37. Fredriksen PM, Hjelle OP, Mamen A, Meza TJ, Westerberg AC. The health Oriented pedagogical project (HOPP) - a controlled longitudinal school-based physical activity intervention program. BMC Public Health. 2017 Apr;17(370):14.

38. Slootmaker SM, Schuit AJ, Chinapaw MJ, Seidell JC, van Mechelen W. Disagreement in physical activity assessed by accelerometer and self-report in subgroups of age, gender, education and weight status. Int J Behav Nutr Phys Act. 2009 Mar 25;6:17.

39. Bell SL, Audrey S, Gunnell D, Cooper A, Campbell R. The relationship between physical activity, mental wellbeing and symptoms of mental health disorder in adolescents: a cohort study. Int J Behav Nutr Phys Act. 2019 Dec 26;16(1):138.

40. Troiano RP, Berrigan D, Dodd KW, Mâsse LC, Tilert T, McDowell M. Physical activity in the United States measured by accelerometer. Med Sci Sports Exerc. 2008 Jan;40(1):181–8.

41. Mattejat F, Remschmidt H, Jozefiak T. ILC - Inventory of Life Quality in Children and Adolescents (Norwegian version by Jozefiak, T. (2012). Hogrefe; 2006.

42. Hogrefe. Hogrefe. 2025 [cited 2025 Jan 17]. ILC Inventory of Life Quality in Children and Adolescents. Available from: https://hogrefe.no/produkt/inventory-of-life-quality-in-children-and-adolescents-ilc/

43. Fredriksen PM, Skår A, Mamen A. Waist circumference in 6-12-year-old children: The Health Oriented Pedagogical Project (HOPP). Scand J Public Health. 2018 May;46(21_suppl):12–20.

Click here to access/download;Figure;Figur1_600dpi.png

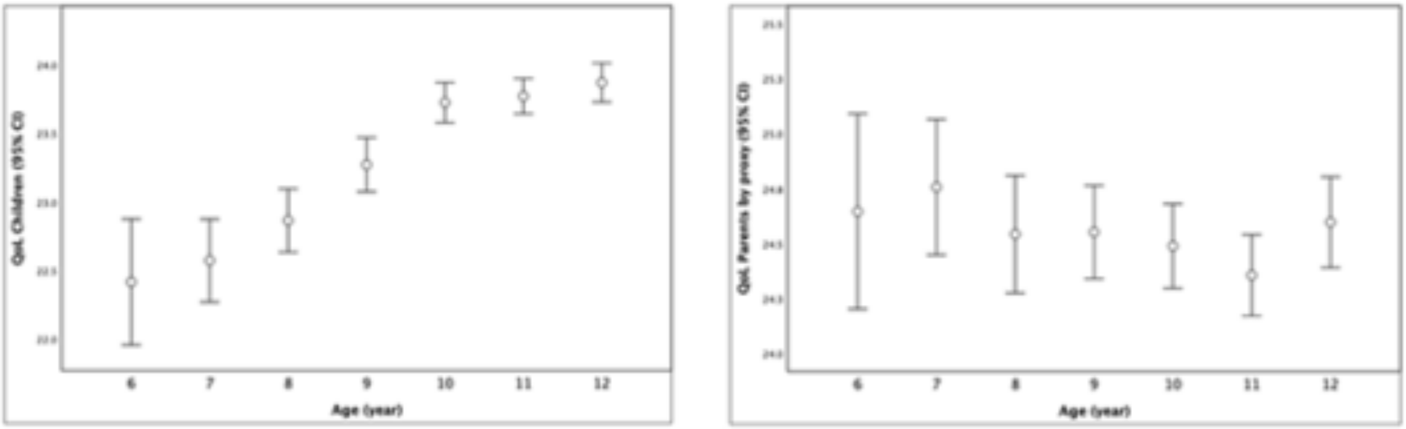

Click here to access/download;Figure;Figur3A_600dpi.png

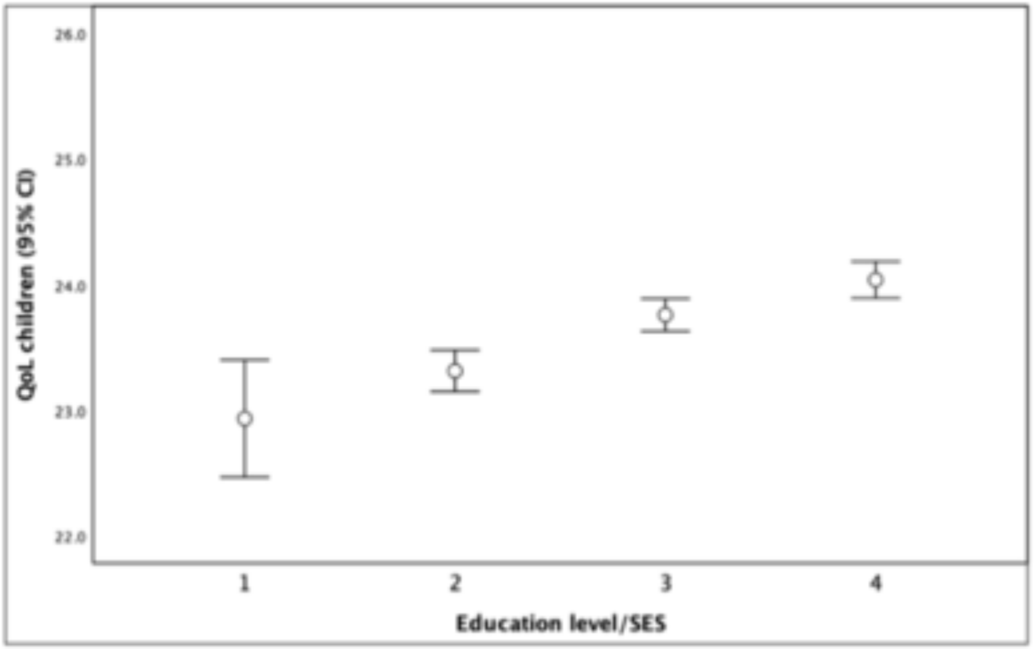

Click here to access/download;Figure;Figur3B_600dpi.png

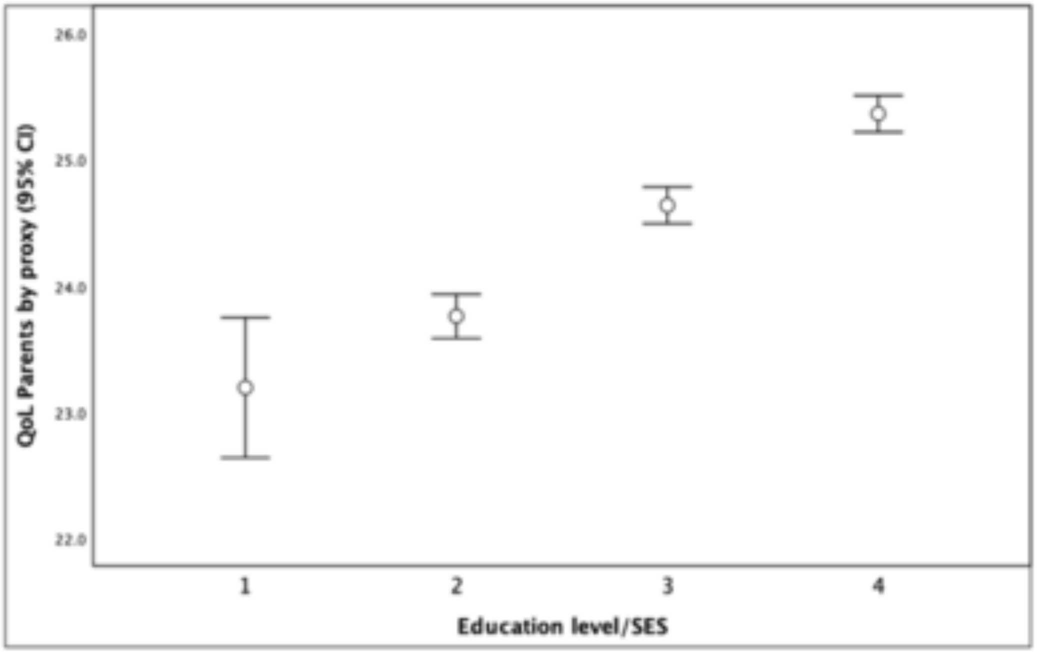

Click here to access/download;Figure;Figure 2 600dpi.PNG

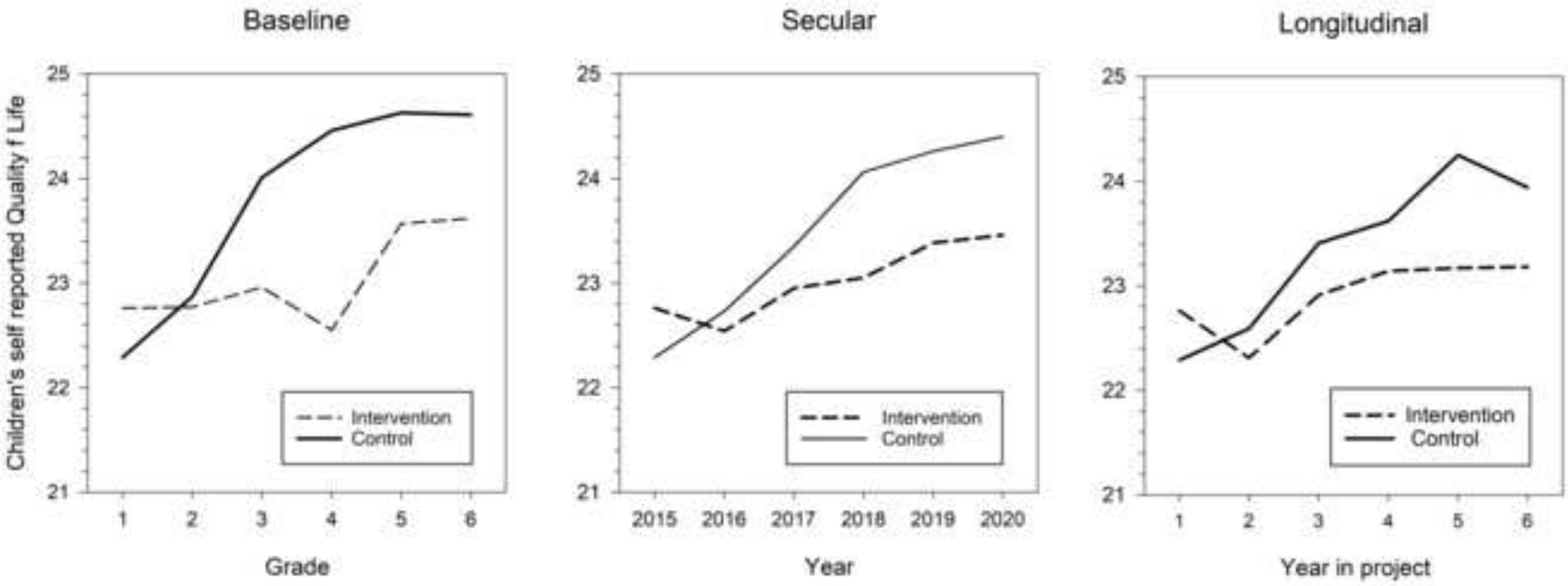

Click here to access/download
Supporting Information
Translation of Regional Ethical Comittee approval.pdf
