# Supplementary material for: Physical activity and quality of life in children: Findings from the Health Oriented Pedagogical Project (HOPP)
Source: PLoS One. 2026 Jul 10;21(7):e0353686. doi: 10.1371/journal.pone.0353686 (PMC13354104; doi:10.1371/journal.pone.0353686)
Supplement: Attachment — Submitted filename: Response to Reviewers.docx [file pone.0353686.s003.docx]

**Response to Reviewers**

Manuscript ID: PONE-D-25-65082

Title: The impact of physical activity on the quality of life in children – the Health Oriented Pedagogical Project (HOPP)

We thank the Academic Editor and the reviewers for their careful evaluation of our manuscript and for the constructive and helpful comments. We have revised the manuscript accordingly and believe that these revisions have improved its clarity and transparency. Below, we provide a point-by-point response to all comments.

**Response to the Academic Editor**

Comment:

Please address minor points: clarify the two groups mentioned in the abstract, provide validity/reliability details for the ILC questionnaire in Methods, and revise the footnotes of Tables 2–3 to clearly indicate the dependent QoL outcomes.

Response:

Thank you for these comments. We have addressed all points as follows:

The abstract has been revised to clearly specify the intervention and control groups.

Information on the validity and reliability of the ILC questionnaire, including references to international and Norwegian validation studies, has been added to the Methods section.

The footnotes of Tables 2 and 3 have been revised to explicitly state that quality of life (QoL) is the dependent outcome variable.

**Response to Reviewer #1**

Comment:

ABSTRACT: Your results suggest two groups. Can you provide additional information about the groups in the methods section of the abstract?

Response:

Yes. The abstract has been revised to explicitly describe the intervention and control groups.

Comment:

METHODS: Could you please provide information about the validity and reliability of the ILC questionnaire?

Response:

Information on the validity and reliability of the ILC questionnaire has now been added to the Methods section, including references to previous international studies and Norwegian validation work.

Comment:

RESULTS: Foot note of tables 2 & 3: Are all variables dependent variables or only QoL? Please clarify or rewrite for clarity.

Response:

The footnotes of Tables 2 and 3 have been revised to clearly indicate that QoL is the dependent outcome variable and that the remaining variables are predictors.

**Response to Reviewer #2**

We thank Reviewer #2 for the thorough and constructive comments and suggestions. We address each point below.

Comment (Line 73):

Please consider eliminating subsections in the introduction section.

Response:

We considered this suggestion. However, we chose to retain the subsections in the Introduction, as they are intended to improve thematic clarity and readability for an interdisciplinary readership, which is consistent with the scope of PLOS ONE.

Comment (Line 77):

Please consider the “p” in italics (also throughout the manuscript).

Response:

All *p*-values are now consistently formatted with the letter *p* in italics.

Comment (Lines 81–97):

Please consider shorter paragraphs to improve readability.

Response:

The manuscript has been revised for readability. Minor adjustments were made where appropriate while maintaining the overall structure and flow of the text.

Comment (Line 107):

Please describe all methodological details, for example, the inclusion and exclusion criteria as well as the associated procedures in detail. Another example, the human resources involved.

Response:

The Methods section provides a detailed description of the study population, recruitment procedures, inclusion criteria, and data collection. Additional methodological details and procedures are described in previously published HOPP protocol papers, which are cited in the manuscript. We therefore believe that the level of methodological detail provided is appropriate for the present article.

Comment (Line 149):

“ILC” previously in full. Please revise the entire manuscript.

Response:

The Inventory of Life Quality in Children and Adolescents (ILC) is now written in full at its first occurrence, and the abbreviation is used consistently throughout the manuscript. One exception is a subsection heading, where the abbreviation is retained for brevity and readability.

Comment (Lines 243 / 281 / 299):

Please revise the tables’ content and format, considering the journal template and instructions for authors.

Response:

Tables 2 and 3 have been revised in accordance with the journal guidelines. In particular, the footnotes have been clarified to explicitly distinguish the dependent outcome variable from the predictor variables.

Comment (Line 314):

It is not clear what the figures’ legends are. Please insert them in the manuscript and consider improving the quality of the figures and not presenting more than one figure together.

Response:

Figure legends have been clarified and are now explicitly included in the manuscript. The figures comply with PLOS ONE guidelines, and multipanel figures were retained where they facilitate comparison and interpretation.

Comment (Line 320):

Please consider improving the quality of the discussion section, particularly with the inclusion of more references.

Response:

The Discussion section has been reviewed to assess whether additional references should be included. We believe that the current set of references appropriately reflects the focus and scope of the study.

Comment (Line 366):

Different format. Please revise the document format details.

Response:

The manuscript has been revised for formatting consistency, and minor adjustments have been made throughout the manuscript. We hope these revisions have improved the clarity and readability of the text.

Comment (Line 427):

Please consider presenting suggestions for future research.

Response:

Suggestions for future research have been clarified in the concluding section of the manuscript.

Comment (Line 428):

Please consider shorter and clearer take-home messages, with practical applications.

Response:

The concluding section has been revised to clarify the main take-home messages and to more explicitly articulate the practical relevance of the findings.

**General comments on language, references, and formatting**

Response:

The manuscript has been carefully reviewed for language, clarity, formatting, and reference consistency in accordance with the journal’s guidelines.
