# Supplementary material for: Physical activity and quality of life in children: Findings from the Health Oriented Pedagogical Project (HOPP)
Source: PLoS One. 2026 Jul 10;21(7):e0353686. doi: 10.1371/journal.pone.0353686 (PMC13354104; doi:10.1371/journal.pone.0353686)
Supplement: Attachment — Submitted filename: Response_to_Reviewers_auresp_2.docx [file pone.0353686.s004.docx]

**Response to Reviewers**

Manuscript ID: PONE-D-25-65082

Title: The impact of physical activity on the quality of life in children – the Health Oriented Pedagogical Project (HOPP)

We thank the Academic Editor and the reviewers for their careful evaluation of our revised manuscript and for their constructive and helpful comments. We have revised the manuscript accordingly and believe that these changes have improved the clarity, transparency, and interpretation of the findings. Below, we provide a point-by-point response to the remaining comments.

**Response to the Academic Editor**

***Comment:* Causal language should be softened**

*Response:*

We agree and have revised the Abstract, Discussion, and Conclusion to consistently avoid causal language. Terms such as “impact” and “effect” have been replaced with “association” or “associated with,” and interpretations have been adjusted accordingly.

***Comment:* Confounding by school-level SES should be better addressed**

*Response:*

We agree and have strengthened the discussion of potential confounding. We now explicitly state that schools were not randomly assigned and differed in socioeconomic context, and that residual confounding at the school or community level cannot be ruled out. We also clarify that group differences should not be interpreted as reflecting causal effects of the intervention.

***Comment:* Interpretation of effect sizes and clinical relevance**

*Response:*
We have expanded the interpretation of effect sizes in both the Results and Discussion. Key regression coefficients are now translated into interpretable contrasts (e.g., change in QoL per 60 minutes of MVPA), and their clinical relevance is discussed in light of the limited magnitude of the observed associations and the high baseline QoL scores.

***Comment:* Clarify primary QoL outcome and ensure consistency**

*Response:*

The primary outcome (LQ0–28) is now clearly defined early in the Methods section and is used consistently throughout the manuscript. The LQ0–100 scale is retained only for descriptive purposes and clearly distinguished from the main analytical outcome.

***Comment:* Minor improvements in tables, intervention description, and missing data reporting**

*Response:*

We have clarified and harmonized table labels and footnotes (Tables 2–3), improved the description of the intervention dose (45 minutes per school day), and added a clear statement in the Statistical analysis section describing how missing data were handled (complete-case analysis, no imputation).

**Response to Reviewer #2**

***Comment:* Confounding by school-level SES and group allocation**

*Response:*

We agree that this is an important limitation. While additional multilevel modelling (e.g., school-level random effects) was considered, we chose not to perform new analyses. We acknowledge that the absence of school-level modelling may have contributed to residual confounding. Instead, we have strengthened the interpretation in the Discussion and Limitations sections. We now explicitly acknowledge that the observed group differences may reflect structural socioeconomic differences between schools rather than intervention effects, and that residual confounding cannot be ruled out.

***Comment:* Interpretation of effect sizes and clinical relevance**

*Response:*

We have revised the manuscript to provide clearer interpretation of effect sizes. For example, the association between MVPA and QoL is now expressed as an approximate increase of 0.36 points per additional 60 minutes of daily MVPA. We further discuss that such differences are small relative to the total scale range and may be of limited clinical relevance, particularly given the high baseline QoL scores.

***Comment:* Ceiling effect**

*Response:*

The discussion of ceiling effects has been expanded in the Limitations section. We now explicitly state that the high baseline QoL scores may have limited the ability to detect meaningful changes or stronger associations over time.

***Comment:* Please consider moving the LQ0–100 transformation to Supplementary Material**

*Response:*

We appreciate this suggestion. The LQ0–100 transformation is not used in the main analyses and is included for descriptive purposes only. We have clarified that LQ0–28 is the primary outcome used throughout the analyses and have streamlined the presentation of the transformation in the Methods section. We retained a brief explanation in the main text to ensure transparency and interpretability for readers. However, we are open to moving additional details to Supplementary Material if preferred by the editor.

***Comment:* Intervention dose and fidelity**

*Response:*
We have clarified the intervention dose throughout the manuscript, specifying that it consisted of approximately 45 minutes of additional physical activity per school day (225 minutes per week). In the Discussion, we now also acknowledge that variability in intensity, engagement, and actual exposure may explain the limited association with QoL.

***Comment:* SES operationalisation**

*Response:*
We have clarified the rationale for using fathers’ education as the SES indicator and report that sensitivity analyses using alternative SES measures (e.g., maternal or highest parental education) yielded similar results. This is now explicitly acknowledged as a limitation.

***Comment:* Missing data and attrition**

*Response:*
We have added a statement in the Statistical analysis section clarifying that missing data were handled using complete-case analyses within the mixed model framework, with no imputation performed. We also note that participant numbers varied over time due to the longitudinal design.

***Comment:* Mixed model specification and covariance structure**

*Response:*
We have clarified the statistical approach in the Methods and Results sections. Alternative repeated covariance structures were evaluated using AIC, and the diagonal structure was retained as it provided the best model fit. This information is now explicitly reported.

***Comment:* Consider splitting Table 1 into separate tables**

*Response:*

We considered splitting Table 1 as suggested. However, we chose to retain the current format, as it allows for a combined presentation of baseline, longitudinal, and secular trends within a single overview.

***Comment:* Non-estimable terms in child-reported models**

*Response:*
We acknowledge this limitation and have clarified it in the Limitations section. We now state that some overall terms were not estimable due to sparse data in specific group-by-year combinations. However, the models were retained because they remained informative for several continuous covariates, including MVPA and age.

We appreciate the reviewer’s and editor’s constructive feedback, which has helped improve the manuscript. We believe that the revisions have strengthened methodological transparency and the interpretation of the findings, and we hope that the manuscript is now suitable for publication.
