# Supplementary material for: Physical activity and quality of life in children: Findings from the Health Oriented Pedagogical Project (HOPP)
Source: PLoS One. 2026 Jul 10;21(7):e0353686. doi: 10.1371/journal.pone.0353686 (PMC13354104; doi:10.1371/journal.pone.0353686)
Supplement: Attachment — Submitted filename: Response to Reviewers 02.docx [file pone.0353686.s005.docx]

Response to Reviewers

Manuscript ID: PONE-D-25-65082R2

Title: The impact of physical activity on the quality of life in children – the Health Oriented Pedagogical Project (HOPP)

We thank the Academic Editor for the additional comments and the opportunity to clarify the relationship between the present manuscript and other HOPP-related submissions currently under consideration at PLOS ONE.

**Response to the Academic Editor**

**Comment:** Please clarify how the present manuscript is scientifically distinct from related HOPP manuscripts currently under consideration at PLOS ONE, including the extent of overlap in study population, dataset, study period, variables, analyses, and findings.

**Response:**

We appreciate the opportunity to clarify this issue.

The present manuscript originates from the Health Oriented Pedagogical Project (HOPP), a large longitudinal school-based cohort study. As is common in longitudinal cohort studies, multiple scientific questions can be investigated using the same overall study population and data collection period. Consequently, some overlap exists in study population, study period, and selected background variables across HOPP-related manuscripts. However, the research aims, outcome variables, analytical approaches, and conclusions are distinct.

At present, two additional HOPP-related manuscripts are under consideration at PLOS ONE. One focuses on **waist-to-height ratio (WHtR)** as the primary outcome, while the second focuses on **metabolic syndrome and cardiometabolic risk factors**. The present manuscript differs from both by examining **quality of life (QoL)**, measured using the Inventory of Life Quality in Children and Adolescents (ILC), as the primary outcome.

The distinctions between the manuscripts can be summarized as follows:

- **Present manuscript (QoL):** Examines associations between QoL, moderate-to-vigorous physical activity (MVPA), socioeconomic status (SES), waist-to-height ratio (WHtR), age, and sex over a five-year period.
- **WHtR manuscript:** Examines determinants and longitudinal development of waist-to-height ratio as the primary outcome.
- **Metabolic syndrome manuscript:** Examines metabolic syndrome and cardiometabolic risk indicators as primary outcomes.

Although the manuscripts share the same underlying cohort and partially overlap in study period and selected explanatory variables, they address different research questions, use different primary outcome measures, and lead to different scientific conclusions.

To further clarify:

- **Study population:** Overlapping HOPP cohort.
- **Study period:** Partially or fully overlapping observation period.
- **Primary outcomes:** QoL (present manuscript), WHtR (related manuscript), and metabolic syndrome/cardiometabolic risk (related manuscript).
- **Research questions:** Distinct and non-overlapping.
- **Statistical analyses:** Different analytical models based on different dependent variables and objectives.
- **Main findings:** Different outcome domains and conclusions.

We confirm that the present manuscript represents an independent scientific contribution. No tables, figures, or analyses are duplicated across the manuscripts. Any overlap is limited to the use of the same cohort and selected background variables, which is typical for longitudinal cohort studies and is fully transparent.

We hope this clarification addresses the editor’s concerns and demonstrates that the manuscript constitutes a distinct and independent publication.

**Best regards,**

Rein Magnus Jensen
On behalf of all authors
