# Supplementary material for: Physical activity and quality of life in children: Findings from the Health Oriented Pedagogical Project (HOPP)
Source: PLoS One. 2026 Jul 10;21(7):e0353686. doi: 10.1371/journal.pone.0353686 (PMC13354104; doi:10.1371/journal.pone.0353686)
Supplement: Peer review history

# Peer review: Decision Letter 0

**DOI:** 10.1371/journal.pone.0353686.r001

**Authors:** Bijit Biswas

27 Jan 2026

PONE-D-25-65082

The impact of physical activity on the quality of life in children – the Health Oriented Pedagogical Project (HOPP)

PLOS One

Dear Dr. Jensen,

Thank you for submitting your manuscript to PLOS ONE. After careful consideration, we feel that it has merit but does not fully meet PLOS ONE’s publication criteria as it currently stands. Therefore, we invite you to submit a revised version of the manuscript that addresses the points raised during the review process.

==============================

**ACADEMIC EDITOR:**  The study is well written and technically sound, with conclusions supported by the data. Please address minor points: clarify the two groups mentioned in the abstract, provide validity/reliability details for the ILC questionnaire in Methods, and revise the footnotes of Tables 2–3 to clearly indicate the dependent QoL outcomes.

Please submit your revised manuscript by Mar 13 2026 11:59PM. If you will need more time than this to complete your revisions, please reply to this message or contact the journal office at plosone@plos.org. When you're ready to submit your revision, log on to https://www.editorialmanager.com/pone/ and select the ’submissions Needing Revision' folder to locate your manuscript file.

Please include the following items when submitting your revised manuscript:

- A letter that responds to each point raised by the academic editor and reviewer(s). You should upload this letter as a separate file labeled 'Response to Reviewers'.
- A marked-up copy of your manuscript that highlights changes made to the original version. You should upload this as a separate file labeled 'Revised Manuscript with Track Changes'.
- An unmarked version of your revised paper without tracked changes. You should upload this as a separate file labeled 'Manuscript'.

-->If you would like to make changes to your financial disclosure, please include your updated statement in your cover letter. Guidelines for resubmitting your figure files are available below the reviewer comments at the end of this letter.

If applicable, we recommend that you deposit your laboratory protocols in protocols.io to enhance the reproducibility of your results. Protocols.io assigns your protocol its own identifier (DOI) so that it can be cited independently in the future. For instructions see: https://journals.plos.org/plosone/s/submission-guidelines#loc-laboratory-protocols. Additionally, PLOS ONE offers an option for publishing peer-reviewed Lab Protocol articles, which describe protocols hosted on protocols.io. Read more information on sharing protocols at https://plos.org/protocols?utm_medium=editorial-email&utm_source=authorletters&utm_campaign=protocols.

We look forward to receiving your revised manuscript.

Kind regards,

Bijit Biswas, MBBS, MD, DNB

Academic Editor

PLOS One

Journal Requirements:

When submitting your revision, we need you to address these additional requirements.

1. Please ensure that your manuscript meets PLOS ONE’s style requirements, including those for file naming. The PLOS ONE style templates can be found at

https://journals.plos.org/plosone/s/file?id=wjVg/PLOSOne_formatting_sample_main_body.pdf and

https://journals.plos.org/plosone/s/file?id=ba62/PLOSOne_formatting_sample_title_authors_affiliations.pdf

2. We note that the grant information you provided in the ‘Funding Information’ and ‘Financial Disclosure’ sections do not match.

When you resubmit, please ensure that you provide the correct grant numbers for the awards you received for your study in the ‘Funding Information’ section

3. Thank you for stating the following financial disclosure:

“The HOPP research project was funded by Horten municipality, Kristiania University College, The Norwegian Order of Odd Fellow Research Fund, The Oslofjord Regional Research Fund, and the Norwegian Fund for Post-Graduate Training in Physiotherapy.”

Please state what role the funders took in the study.  If the funders had no role, please state: "The funders had no role in study design, data collection and analysis, decision to publish, or preparation of the manuscript."

If this statement is not correct you must amend it as needed.

Please include this amended Role of Funder statement in your cover letter; we will change the online submission form on your behalf.

4. Your ethics statement should only appear in the Methods section of your manuscript. If your ethics statement is written in any section besides the Methods, please delete it from any other section.

5. Please include captions for your Supporting Information files at the end of your manuscript, and update any in-text citations to match accordingly. Please see our Supporting Information guidelines for more information: http://journals.plos.org/plosone/s/supporting-information.

6. If the reviewer comments include a recommendation to cite specific previously published works, please review and evaluate these publications to determine whether they are relevant and should be cited. There is no requirement to cite these works unless the editor has indicated otherwise.

Please review your reference list to ensure that it is complete and correct. If you have cited papers that have been retracted, please include the rationale for doing so in the manuscript text, or remove these references and replace them with relevant current references. Any changes to the reference list should be mentioned in the rebuttal letter that accompanies your revised manuscript. If you need to cite a retracted article, indicate the article’s retracted status in the References list and also include a citation and full reference for the retraction notice.

Additional Editor Comments:

The manuscript is generally well written, technically sound, and the conclusions are supported by the presented data. However, a few minor clarifications are required before the work can be considered for acceptance: (1) the abstract mentions two groups, so please briefly specify these groups in the methods/results portion of the abstract for clarity; (2) in the Methods section, kindly provide information on the validity and reliability (or prior validation references) of the ILC questionnaire used to assess quality of life; and (3) please revise or clarify the footnotes of Tables 2 and 3 to indicate whether all listed variables are dependent outcomes or whether only QoL measures are treated as dependent variables. Addressing these points will improve transparency and readability.

[Note: HTML markup is below. Please do not edit.]

Reviewers' comments:

Reviewer’s Responses to Questions

**Comments to the Author**

1. Is the manuscript technically sound, and do the data support the conclusions?

The manuscript must describe a technically sound piece of scientific research with data that supports the conclusions. Experiments must have been conducted rigorously, with appropriate controls, replication, and sample sizes. The conclusions must be drawn appropriately based on the data presented.

Reviewer #1: Yes

Reviewer #2: Partly

**********

2. Has the statistical analysis been performed appropriately and rigorously?

Reviewer #1: Yes

Reviewer #2: Yes

**********

3. Have the authors made all data underlying the findings in their manuscript fully available?

The PLOS Data policy requires authors to make all data underlying the findings described in their manuscript fully available without restriction, with rare exception (please refer to the Data Availability Statement in the manuscript PDF file). The data should be provided as part of the manuscript or its supporting information, or deposited to a public repository. For example, in addition to summary statistics, the data points behind means, medians and variance measures should be available. If there are restrictions on publicly sharing data—e.g. participant privacy or use of data from a third party—those must be specified.

Reviewer #1: Yes

Reviewer #2: Yes

**********

4. Is the manuscript presented in an intelligible fashion and written in standard English?

PLOS ONE does not copyedit accepted manuscripts, so the language in submitted articles must be clear, correct, and unambiguous. Any typographical or grammatical errors should be corrected at revision, so please note any specific errors here.

Reviewer #1: Yes

Reviewer #2: Yes

**********

5. Review Comments to the Author

Please use the space provided to explain your answers to the questions above. You may also include additional comments for the author, including concerns about dual publication, research ethics, or publication ethics. (Please upload your review as an attachment if it exceeds 20,000 characters)

Reviewer #1: The study assessed the impact of physical activity on quality of life among children. It is well written. Minor comments for the authors to address.

ABSTRACT: Your results suggest two groups. Can you provide additional information about the groups in the methods section of the abstract?

METHODS: Could you please provide information about the validity and reliability of the ILC questionnaire?

RESULTS

Foot note of tables 2 & 3: Are all variables dependent variables or only QoL? Please clarify or rewrite for clarity.

Reviewer #2: Dear Authors,

I want to express my gratitude for the opportunity to revise this manuscript.

The document is globally well-organized and well-written.

Below are suggestions with line indication:

73 - Please consider eliminating subsections in the introduction section.

77 - Please consider the “p” in italics (also throughout the manuscript – e.g. L223).

81-97 - Please consider shorter paragraphs to improve readability (8-12 lines suggested). Please consider this suggestion throughout the manuscript.

107 - Please describe all methodological details, for example, the inclusion and exclusion criteria as well as the associated procedures in detail. Another example, the human resources involved (academic background and experience), preferably with reference support.

149 – “ILC” previously in full. Please revise the entire manuscript.

243 / 281 / 299 – Please revise the tables´ content and format, considering the journal template and instructions for authors.

314 – It is not clear what the figures´ legends are, please insert them in the manuscript. Moreover, please consider improving the quality of the figures and not presenting more than one figure together to improve data interpretation conditions.

320 - Please consider improving the quality of the discussion section, particularly with the inclusion of more references.

366 – Different format. Please revise the document format details.

427 – Please consider presenting suggestions for future research,

428 - Please consider shorter and clearer take-home messages, if possible, with practical applications.

Please revise and standardize the references, considering the journal template and instructions for authors.

Please carefully revise the English details and document format, considering the journal template and instructions for authors.

**********

6. PLOS authors have the option to publish the peer review history of their article (what does this mean?). If published, this will include your full peer review and any attached files.

If you choose “no”, your identity will remain anonymous but your review may still be made public.

**Do you want your identity to be public for this peer review?**  For information about this choice, including consent withdrawal, please see our Privacy Policy.

Reviewer #1: No

Reviewer #2: No

**********

[NOTE: If reviewer comments were submitted as an attachment file, they will be attached to this email and accessible via the submission site. Please log into your account, locate the manuscript record, and check for the action link "View Attachments". If this link does not appear, there are no attachment files.]

To ensure your figures meet our technical requirements, please review our figure guidelines: https://journals.plos.org/plosone/s/figures

You may also use PLOS’s free figure tool, NAAS, to help you prepare publication quality figures: https://journals.plos.org/plosone/s/figures#loc-tools-for-figure-preparation.

NAAS will assess whether your figures meet our technical requirements by comparing each figure against our figure specifications.

Attachment Submitted filename: PONE-D-25-65082.pdf

# Author comment: Author response to Decision Letter 1

**DOI:** 10.1371/journal.pone.0353686.r002

13 Feb 2026

Response to the Academic Editor

Comment:

Please address minor points: clarify the two groups mentioned in the abstract, provide validity/reliability details for the ILC questionnaire in Methods, and revise the footnotes of Tables 2–3 to clearly indicate the dependent QoL outcomes.

Response:

Thank you for these comments. We have addressed all points as follows:

The abstract has been revised to clearly specify the intervention and control groups.

Information on the validity and reliability of the ILC questionnaire, including references to international and Norwegian validation studies, has been added to the Methods section.

The footnotes of Tables 2 and 3 have been revised to explicitly state that quality of life (QoL) is the dependent outcome variable.

Response to Reviewer #1

Comment:

ABSTRACT: Your results suggest two groups. Can you provide additional information about the groups in the methods section of the abstract?

Response:

Yes. The abstract has been revised to explicitly describe the intervention and control groups.

Comment:

METHODS: Could you please provide information about the validity and reliability of the ILC questionnaire?

Response:

Information on the validity and reliability of the ILC questionnaire has now been added to the Methods section, including references to previous international studies and Norwegian validation work.

Comment:

RESULTS: Foot note of tables 2 & 3: Are all variables dependent variables or only QoL? Please clarify or rewrite for clarity.

Response:

The footnotes of Tables 2 and 3 have been revised to clearly indicate that QoL is the dependent outcome variable and that the remaining variables are predictors.

Response to Reviewer #2

We thank Reviewer #2 for the thorough and constructive comments and suggestions. We address each point below.

Comment (Line 73):

Please consider eliminating subsections in the introduction section.

Response:

We considered this suggestion. However, we chose to retain the subsections in the Introduction, as they are intended to improve thematic clarity and readability for an interdisciplinary readership, which is consistent with the scope of PLOS ONE.

Comment (Line 77):

Please consider the “p” in italics (also throughout the manuscript).

Response:

All p-values are now consistently formatted with the letter p in italics.

Comment (Lines 81–97):

Please consider shorter paragraphs to improve readability.

Response:

The manuscript has been revised for readability. Minor adjustments were made where appropriate while maintaining the overall structure and flow of the text.

Comment (Line 107):

Please describe all methodological details, for example, the inclusion and exclusion criteria as well as the associated procedures in detail. Another example, the human resources involved.

Response:

The Methods section provides a detailed description of the study population, recruitment procedures, inclusion criteria, and data collection. Additional methodological details and procedures are described in previously published HOPP protocol papers, which are cited in the manuscript. We therefore believe that the level of methodological detail provided is appropriate for the present article.

Comment (Line 149):

“ILC” previously in full. Please revise the entire manuscript.

Response:

The Inventory of Life Quality in Children and Adolescents (ILC) is now written in full at its first occurrence, and the abbreviation is used consistently throughout the manuscript. One exception is a subsection heading, where the abbreviation is retained for brevity and readability.

Comment (Lines 243 / 281 / 299):

Please revise the tables’ content and format, considering the journal template and instructions for authors.

Response:

Tables 2 and 3 have been revised in accordance with the journal guidelines. In particular, the footnotes have been clarified to explicitly distinguish the dependent outcome variable from the predictor variables.

Comment (Line 314):

It is not clear what the figures’ legends are. Please insert them in the manuscript and consider improving the quality of the figures and not presenting more than one figure together.

Response:

Figure legends have been clarified and are now explicitly included in the manuscript. The figures comply with PLOS ONE guidelines, and multipanel figures were retained where they facilitate comparison and interpretation.

Comment (Line 320):

Please consider improving the quality of the discussion section, particularly with the inclusion of more references.

Response:

The Discussion section has been reviewed to assess whether additional references should be included. We believe that the current set of references appropriately reflects the focus and scope of the study.

Comment (Line 366):

Different format. Please revise the document format details.

Response:

The manuscript has been revised for formatting consistency, and minor adjustments have been made throughout the manuscript. We hope these revisions have improved the clarity and readability of the text.

Comment (Line 427):

Please consider presenting suggestions for future research.

Response:

Suggestions for future research have been clarified in the concluding section of the manuscript.

Comment (Line 428):

Please consider shorter and clearer take-home messages, with practical applications.

Response:

The concluding section has been revised to clarify the main take-home messages and to more explicitly articulate the practical relevance of the findings.

General comments on language, references, and formatting

Response:

The manuscript has been carefully reviewed for language, clarity, formatting, and reference consistency in accordance with the journal’s guidelines.

Attachment Submitted filename: Response to Reviewers.docx

# Peer review: Decision Letter 1

**DOI:** 10.1371/journal.pone.0353686.r003

**Authors:** Bijit Biswas, Bijit Biswas

4 Mar 2026

PONE-D-25-65082R1

The impact of physical activity on the quality of life in children – the Health Oriented Pedagogical Project (HOPP)

PLOS One

Dear Dr. Jensen,

Thank you for submitting your manuscript to PLOS ONE. After careful consideration, we feel that it has merit but does not fully meet PLOS ONE’s publication criteria as it currently stands. Therefore, we invite you to submit a revised version of the manuscript that addresses the points raised during the review process.

Please submit your revised manuscript by Apr 18 2026 11:59PM. If you will need more time than this to complete your revisions, please reply to this message or contact the journal office at plosone@plos.org. When you're ready to submit your revision, log on to https://www.editorialmanager.com/pone/ and select the ’submissions Needing Revision' folder to locate your manuscript file.

Please include the following items when submitting your revised manuscript:

- A letter that responds to each point raised by the academic editor and reviewer(s). You should upload this letter as a separate file labeled 'Response to Reviewers'.
- A marked-up copy of your manuscript that highlights changes made to the original version. You should upload this as a separate file labeled 'Revised Manuscript with Track Changes'.
- An unmarked version of your revised paper without tracked changes. You should upload this as a separate file labeled 'Manuscript'.

If you would like to make changes to your financial disclosure, please include your updated statement in your cover letter. Guidelines for resubmitting your figure files are available below the reviewer comments at the end of this letter.

If applicable, we recommend that you deposit your laboratory protocols in protocols.io to enhance the reproducibility of your results. Protocols.io assigns your protocol its own identifier (DOI) so that it can be cited independently in the future. For instructions see: https://journals.plos.org/plosone/s/submission-guidelines#loc-laboratory-protocols. Additionally, PLOS ONE offers an option for publishing peer-reviewed Lab Protocol articles, which describe protocols hosted on protocols.io. Read more information on sharing protocols at https://plos.org/protocols?utm_medium=editorial-email&utm_source=authorletters&utm_campaign=protocols.

We look forward to receiving your revised manuscript.

Kind regards,

Bijit Biswas, MBBS, MD, DNB

Academic Editor

PLOS One

**Journal Requirements:**

1. If the reviewer comments include a recommendation to cite specific previously published works, please review and evaluate these publications to determine whether they are relevant and should be cited. There is no requirement to cite these works unless the editor has indicated otherwise.

**Additional Editor Comments:**

The manuscript presents an important long-term school-based physical activity intervention. While many previous comments appear to have been addressed, several issues still require clarification. The causal language should be softened given the study design, and potential confounding by school-level SES should be better addressed in the analyses and discussion. The primary QoL outcome should be clearly defined and used consistently, and effect sizes should be interpreted in a more clinically meaningful way. Minor improvements in table presentation, intervention description, and reporting of missing data would further enhance clarity.

[Note: HTML markup is below. Please do not edit.]

Reviewers' comments:

Reviewer’s Responses to Questions

**Comments to the Author**

1. If the authors have adequately addressed your comments raised in a previous round of review and you feel that this manuscript is now acceptable for publication, you may indicate that here to bypass the “Comments to the Author” section, enter your conflict of interest statement in the “Confidential to Editor” section, and submit your "Accept" recommendation.

Reviewer #1: All comments have been addressed

Reviewer #2: All comments have been addressed

**********

2. Is the manuscript technically sound, and do the data support the conclusions?

The manuscript must describe a technically sound piece of scientific research with data that supports the conclusions. Experiments must have been conducted rigorously, with appropriate controls, replication, and sample sizes. The conclusions must be drawn appropriately based on the data presented.

Reviewer #1: (No Response)

Reviewer #2: Partly

**********

3. Has the statistical analysis been performed appropriately and rigorously?

Reviewer #1: (No Response)

Reviewer #2: Yes

**********

4. Have the authors made all data underlying the findings in their manuscript fully available?

The PLOS Data policy requires authors to make all data underlying the findings described in their manuscript fully available without restriction, with rare exception (please refer to the Data Availability Statement in the manuscript PDF file). The data should be provided as part of the manuscript or its supporting information, or deposited to a public repository. For example, in addition to summary statistics, the data points behind means, medians and variance measures should be available. If there are restrictions on publicly sharing data—e.g. participant privacy or use of data from a third party—those must be specified.

Reviewer #1: (No Response)

Reviewer #2: Yes

**********

5. Is the manuscript presented in an intelligible fashion and written in standard English?

PLOS ONE does not copyedit accepted manuscripts, so the language in submitted articles must be clear, correct, and unambiguous. Any typographical or grammatical errors should be corrected at revision, so please note any specific errors here.

Reviewer #1: (No Response)

Reviewer #2: Yes

**********

6. Review Comments to the Author

Please use the space provided to explain your answers to the questions above. You may also include additional comments for the author, including concerns about dual publication, research ethics, or publication ethics. (Please upload your review as an attachment if it exceeds 20,000 characters)

Reviewer #1: (No Response)

Reviewer #2: Dear Authors,

I want to express my gratitude for the opportunity to revise this manuscript.

The manuscript reports a large, 5‑year Norwegian school-based physical activity (PA) intervention (HOPP) in nine primary schools, examining how objectively measured PA, waist‑to‑height ratio (WHtR) and socioeconomic status (SES, fathers’ education) relate to children’s quality of life (QoL, ILC) from ages 6–12 years.

Below are suggestions:

Major issues (with section/line indications)

Causal language vs. design and analyses

In the Abstract and Conclusion, soften causal wording (e.g., “impact,” “effect”) unless you demonstrate that changes over time between groups are attributable to the intervention beyond SES differences.

Confounding by school‑level SES and group allocation

Please consider Including school (or municipality) as a random effect and/or adjust explicitly for school‑level SES indicators if available, and report how this affects the β for intervention vs control.

In the Discussion, more clearly distinguish what can be attributed to the intervention from what likely reflects structural SES differences.

Interpretation of effect sizes and clinical relevance

Please consider translate key β coefficients into interpretable contrasts (e.g., change in QoL per 60 minutes additional MVPA per day, or per shift from WHtR 0.45 to 0.55) and discuss whether these differences are likely clinically meaningful.

In Limitations, expand the discussion of the ceiling effect and how it constrains detectability of such small associations.

Outcome definition and use of different QoL metrics

Please clearly state early in Methods which QoL metric is the primary outcome (e.g., LQ0–28) and use that consistently throughout the paper, including Abstract, Results, and Discussion.

Please consider moving the LQ0–100 transformation to Supplementary Material unless it is directly used in analyses.

Presentation and clarity of Tables and Figures

Consider splitting Table 1 into separate tables or moving part of it to Supplementary Material, keeping in the main text only the essential descriptive results.

Please harmonize labels across Tables 2–3 and their footnotes; define all abbreviations once and simplify the explanatory footnotes to focus on the dependent variable and set of predictors.

Rationale and fidelity for the intervention

Add a concise summary (with numbers) of MVPA differences between groups over time (or reference specific previously published HOPP results and clearly state what was observed in this cohort), to support that an actual PA contrast existed.

Briefly discuss whether the limited effect on QoL could reflect insufficient PA intensity, duration, or engagement, despite the nominal 225 minutes.

Use of parental SES proxy (father’s education only)

Justify the choice of father’s education vs either parent/highest parental education and, if data exist, present sensitivity analyses using mothers’ or highest parental education.

Acknowledge this as a limitation in the Limitations section.

Minor issues (with section/line indications)

Terminology consistency (PA/MVPA, SES definition)

In Abstract Results, specify “MVPA” rather than generic PA, and ensure “SES: father’s education level” is grammatically corrected.

Grammar and style edits

Conduct a careful language edit for minor typos, missing articles, and punctuation.

Clarify dose description of PA in intervention

Specify whether “45 minutes” refers to daily added PA and make it consistent across Methods and Limitations.

Outcome scale explanation could be streamlined

Keep a short explanation and move the worked example (24/28 × 100 = 85.7) to Supplementary Material.

Clarify baseline age and grade coverage

Add a short sentence clarifying grade–age mapping at baseline (e.g., “Grade 1 ≈ 6–7 years, … grade 6 ≈ 11–12 years”).

Clarify how missing data and attrition were handled

Add a sentence in Statistical analysis describing how missing QoL, SES, PA, and WHtR data were treated and whether any sensitivity analyses were performed.

Figure axis and labelling clarity

Rephrase as “the Y‑axis was narrowed (e.g., 20–26) to better visualise small differences” and ensure axis ranges are clearly shown.

Redundant repetition of track-change notes

Ensure all editorial marks (e.g., “Formatted: Font: Italic”) are removed from the final submission.

Clarify dual data availability statement

Clarify whether all analytic data are in the public repository, and if not, specify which subset is publicly available and which require request.

Reference cross‑checking

Check that each in‑text citation range matches the specific claim (e.g., meta-analysis on PA and QoL vs WHO PA guidelines) and split ranges if needed.

**********

7. PLOS authors have the option to publish the peer review history of their article (what does this mean?). If published, this will include your full peer review and any attached files.

If you choose “no”, your identity will remain anonymous but your review may still be made public.

**Do you want your identity to be public for this peer review?**  For information about this choice, including consent withdrawal, please see our Privacy Policy.

Reviewer #1: No

Reviewer #2: No

**********

[NOTE: If reviewer comments were submitted as an attachment file, they will be attached to this email and accessible via the submission site. Please log into your account, locate the manuscript record, and check for the action link "View Attachments". If this link does not appear, there are no attachment files.]

To ensure your figures meet our technical requirements, please review our figure guidelines: https://journals.plos.org/plosone/s/figures

You may also use PLOS’s free figure tool, NAAS, to help you prepare publication quality figures: https://journals.plos.org/plosone/s/figures#loc-tools-for-figure-preparation.

NAAS will assess whether your figures meet our technical requirements by comparing each figure against our figure specifications.

# Author comment: Author response to Decision Letter 2

**DOI:** 10.1371/journal.pone.0353686.r004

18 Apr 2026

Response to Reviewers

Manuscript ID: PONE-D-25-65082

Title: The impact of physical activity on the quality of life in children – the Health Oriented Pedagogical Project (HOPP)

We thank the Academic Editor and the reviewers for their careful evaluation of our revised manuscript and for their constructive and helpful comments. We have revised the manuscript accordingly and believe that these changes have improved the clarity, transparency, and interpretation of the findings. Below, we provide a point-by-point response to the remaining comments.

Response to the Academic Editor

Comment: Causal language should be softened

Response:

We agree and have revised the Abstract, Discussion, and Conclusion to consistently avoid causal language. Terms such as “impact” and “effect” have been replaced with “association” or “associated with,” and interpretations have been adjusted accordingly.

Comment: Confounding by school-level SES should be better addressed

Response:

We agree and have strengthened the discussion of potential confounding. We now explicitly state that schools were not randomly assigned and differed in socioeconomic context, and that residual confounding at the school or community level cannot be ruled out. We also clarify that group differences should not be interpreted as reflecting causal effects of the intervention.

Comment: Interpretation of effect sizes and clinical relevance

Response:

We have expanded the interpretation of effect sizes in both the Results and Discussion. Key regression coefficients are now translated into interpretable contrasts (e.g., change in QoL per 60 minutes of MVPA), and their clinical relevance is discussed in light of the limited magnitude of the observed associations and the high baseline QoL scores.

Comment: Clarify primary QoL outcome and ensure consistency

Response:

The primary outcome (LQ0–28) is now clearly defined early in the Methods section and is used consistently throughout the manuscript. The LQ0–100 scale is retained only for descriptive purposes and clearly distinguished from the main analytical outcome.

Comment: Minor improvements in tables, intervention description, and missing data reporting

Response:

We have clarified and harmonized table labels and footnotes (Tables 2–3), improved the description of the intervention dose (45 minutes per school day), and added a clear statement in the Statistical analysis section describing how missing data were handled (complete-case analysis, no imputation).

Response to Reviewer #2

Comment: Confounding by school-level SES and group allocation

Response:

We agree that this is an important limitation. While additional multilevel modelling (e.g., school-level random effects) was considered, we chose not to perform new analyses. We acknowledge that the absence of school-level modelling may have contributed to residual confounding. Instead, we have strengthened the interpretation in the Discussion and Limitations sections. We now explicitly acknowledge that the observed group differences may reflect structural socioeconomic differences between schools rather than intervention effects, and that residual confounding cannot be ruled out.

Comment: Interpretation of effect sizes and clinical relevance

Response:

We have revised the manuscript to provide clearer interpretation of effect sizes. For example, the association between MVPA and QoL is now expressed as an approximate increase of 0.36 points per additional 60 minutes of daily MVPA. We further discuss that such differences are small relative to the total scale range and may be of limited clinical relevance, particularly given the high baseline QoL scores.

Comment: Ceiling effect

Response:

The discussion of ceiling effects has been expanded in the Limitations section. We now explicitly state that the high baseline QoL scores may have limited the ability to detect meaningful changes or stronger associations over time.

Comment: Please consider moving the LQ0–100 transformation to Supplementary Material

Response:

We appreciate this suggestion. The LQ0–100 transformation is not used in the main analyses and is included for descriptive purposes only. We have clarified that LQ0–28 is the primary outcome used throughout the analyses and have streamlined the presentation of the transformation in the Methods section. We retained a brief explanation in the main text to ensure transparency and interpretability for readers. However, we are open to moving additional details to Supplementary Material if preferred by the editor.

Comment: Intervention dose and fidelity

Response:

We have clarified the intervention dose throughout the manuscript, specifying that it consisted of approximately 45 minutes of additional physical activity per school day (225 minutes per week). In the Discussion, we now also acknowledge that variability in intensity, engagement, and actual exposure may explain the limited association with QoL.

Comment: SES operationalisation

Response:

We have clarified the rationale for using fathers’ education as the SES indicator and report that sensitivity analyses using alternative SES measures (e.g., maternal or highest parental education) yielded similar results. This is now explicitly acknowledged as a limitation.

Comment: Missing data and attrition

Response:

We have added a statement in the Statistical analysis section clarifying that missing data were handled using complete-case analyses within the mixed model framework, with no imputation performed. We also note that participant numbers varied over time due to the longitudinal design.

Comment: Mixed model specification and covariance structure

Response:

We have clarified the statistical approach in the Methods and Results sections. Alternative repeated covariance structures were evaluated using AIC, and the diagonal structure was retained as it provided the best model fit. This information is now explicitly reported.

Comment: Consider splitting Table 1 into separate tables

Response:

We considered splitting Table 1 as suggested. However, we chose to retain the current format, as it allows for a combined presentation of baseline, longitudinal, and secular trends within a single overview.

Comment: Non-estimable terms in child-reported models

Response:

We acknowledge this limitation and have clarified it in the Limitations section. We now state that some overall terms were not estimable due to sparse data in specific group-by-year combinations. However, the models were retained because they remained informative for several continuous covariates, including MVPA and age.

We appreciate the reviewer’s and editor’s constructive feedback, which has helped improve the manuscript. We believe that the revisions have strengthened methodological transparency and the interpretation of the findings, and we hope that the manuscript is now suitable for publication.

Attachment Submitted filename: Response_to_Reviewers_auresp_2.docx

# Peer review: Decision Letter 2

**DOI:** 10.1371/journal.pone.0353686.r005

**Authors:** Bijit Biswas, Bijit Biswas, Bijit Biswas

30 May 2026

PONE-D-25-65082R2

The impact of physical activity on the quality of life in children – the Health Oriented Pedagogical Project (HOPP)

PLOS One

Dear Dr.  Jensen,

Thank you for submitting your manuscript to PLOS ONE. After careful consideration, we feel that it has merit but does not fully meet PLOS ONE’s publication criteria as it currently stands. Therefore, we invite you to submit a revised version of the manuscript that addresses the points raised during the review process.

Please submit your revised manuscript by Jul 14 2026 11:59PM. If you will need more time than this to complete your revisions, please reply to this message or contact the journal office at plosone@plos.org. When you're ready to submit your revision, log on to https://www.editorialmanager.com/pone/ and select the ’submissions Needing Revision' folder to locate your manuscript file.

Please include the following items when submitting your revised manuscript:

- A letter that responds to each point raised by the academic editor and reviewer(s). You should upload this letter as a separate file labeled 'Response to Reviewers'.
- A marked-up copy of your manuscript that highlights changes made to the original version. You should upload this as a separate file labeled 'Revised Manuscript with Track Changes'.
- An unmarked version of your revised paper without tracked changes. You should upload this as a separate file labeled 'Manuscript'.

If you would like to make changes to your financial disclosure, please include your updated statement in your cover letter. Guidelines for resubmitting your figure files are available below the reviewer comments at the end of this letter.

If applicable, we recommend that you deposit your laboratory protocols in protocols.io to enhance the reproducibility of your results. Protocols.io assigns your protocol its own identifier (DOI) so that it can be cited independently in the future. For instructions see: https://journals.plos.org/plosone/s/submission-guidelines#loc-laboratory-protocols. Additionally, PLOS ONE offers an option for publishing peer-reviewed Lab Protocol articles, which describe protocols hosted on protocols.io. Read more information on sharing protocols at https://plos.org/protocols?utm_medium=editorial-email&utm_source=authorletters&utm_campaign=protocols.

As the corresponding author, your ORCID iD is verified in the submission system and will appear in the published article. PLOS supports the use of ORCID, and we encourage all coauthors to register for an ORCID iD and use it as well. Please encourage your coauthors to verify their ORCID iD within the submission system before final acceptance, as unverified ORCID iDs will not appear in the published article. *Only*  the individual author can complete the verification step; PLOS staff *cannot*  verify ORCID iDs on behalf of authors.

We look forward to receiving your revised manuscript.

Kind regards,

Bijit Biswas, MBBS, MD, DNB

Academic Editor

PLOS One

Journal Requirements:

1. If the reviewer comments include a recommendation to cite specific previously published works, please review and evaluate these publications to determine whether they are relevant and should be cited. There is no requirement to cite these works unless the editor has indicated otherwise.

Additional Editor Comments:

Thank you for submitting the revised manuscript. The reviewers' comments have been satisfactorily addressed. However, as informed by the journal staff, related manuscripts arising from the Health Oriented Pedagogical Project (HOPP) are currently under consideration at PLOS ONE. In accordance with the journal’s policy on related submissions, please clarify how the present manuscript is scientifically distinct from these submissions, including the extent of overlap in study population, dataset, study period, variables, analyses, and findings. Please also confirm that the manuscript can be interpreted as a fully independent publication and identify any overlapping text, tables, figures, or analyses. In addition, given that this study is based on data collected as part of a registered clinical trial (NCT02495714), the manuscript may be subject to independent statistical review. Please ensure that the statistical methods, analytical approach, handling of missing data, and interpretation of results are clearly described to facilitate further evaluation. If substantial overlap exists and the distinction between the manuscripts is not sufficiently clear, the authors may be advised to combine the related submissions into a single comprehensive publication in accordance with PLOS ONE policy.

[Note: HTML markup is below. Please do not edit.]

Reviewer’s Responses to Questions

**Comments to the Author**

1. If the authors have adequately addressed your comments raised in a previous round of review and you feel that this manuscript is now acceptable for publication, you may indicate that here to bypass the “Comments to the Author” section, enter your conflict of interest statement in the “Confidential to Editor” section, and submit your "Accept" recommendation.

Reviewer #2: All comments have been addressed

**********

2. Is the manuscript technically sound, and do the data support the conclusions?

The manuscript must describe a technically sound piece of scientific research with data that supports the conclusions. Experiments must have been conducted rigorously, with appropriate controls, replication, and sample sizes. The conclusions must be drawn appropriately based on the data presented.

Reviewer #2: Yes

**********

3. Has the statistical analysis been performed appropriately and rigorously?

Reviewer #2: Yes

**********

4. Have the authors made all data underlying the findings in their manuscript fully available?

The PLOS Data policy requires authors to make all data underlying the findings described in their manuscript fully available without restriction, with rare exception (please refer to the Data Availability Statement in the manuscript PDF file). The data should be provided as part of the manuscript or its supporting information, or deposited to a public repository. For example, in addition to summary statistics, the data points behind means, medians and variance measures should be available. If there are restrictions on publicly sharing data—e.g. participant privacy or use of data from a third party—those must be specified.

Reviewer #2: Yes

**********

5. Is the manuscript presented in an intelligible fashion and written in standard English?

PLOS ONE does not copyedit accepted manuscripts, so the language in submitted articles must be clear, correct, and unambiguous. Any typographical or grammatical errors should be corrected at revision, so please note any specific errors here.

Reviewer #2: Yes

**********

6. Review Comments to the Author

Please use the space provided to explain your answers to the questions above. You may also include additional comments for the author, including concerns about dual publication, research ethics, or publication ethics. (Please upload your review as an attachment if it exceeds 20,000 characters)

Reviewer #2: Dear Authors,

I would like to extend my heartfelt thanks for your valuable contributions during the review process and to congratulate you all on the work you've done on this article.

Best wishes for continued excellent work.

Thank you.

Best regards.

**********

7. PLOS authors have the option to publish the peer review history of their article (what does this mean?). If published, this will include your full peer review and any attached files.

If you choose “no”, your identity will remain anonymous but your review may still be made public.

**Do you want your identity to be public for this peer review?**  For information about this choice, including consent withdrawal, please see our Privacy Policy.

Reviewer #2: No

**********

[NOTE: If reviewer comments were submitted as an attachment file, they will be attached to this email and accessible via the submission site. Please log into your account, locate the manuscript record, and check for the action link "View Attachments". If this link does not appear, there are no attachment files.]

To ensure your figures meet our technical requirements, please review our figure guidelines: https://journals.plos.org/plosone/s/figures

You may also use PLOS’s free figure tool, NAAS, to help you prepare publication quality figures: https://journals.plos.org/plosone/s/figures#loc-tools-for-figure-preparation.

NAAS will assess whether your figures meet our technical requirements by comparing each figure against our figure specifications.

# Author comment: Author response to Decision Letter 3

**DOI:** 10.1371/journal.pone.0353686.r006

4 Jun 2026

Response to Reviewers

Manuscript ID: PONE-D-25-65082R2

Title: The impact of physical activity on the quality of life in children – the Health Oriented Pedagogical Project (HOPP)

We thank the Academic Editor for the additional comments and the opportunity to clarify the relationship between the present manuscript and other HOPP-related submissions currently under consideration at PLOS ONE.

Response to the Academic Editor

Comment: Please clarify how the present manuscript is scientifically distinct from related HOPP manuscripts currently under consideration at PLOS ONE, including the extent of overlap in study population, dataset, study period, variables, analyses, and findings.

Response:

We appreciate the opportunity to clarify this issue.

The present manuscript originates from the Health Oriented Pedagogical Project (HOPP), a large longitudinal school-based cohort study. As is common in longitudinal cohort studies, multiple scientific questions can be investigated using the same overall study population and data collection period. Consequently, some overlap exists in study population, study period, and selected background variables across HOPP-related manuscripts. However, the research aims, outcome variables, analytical approaches, and conclusions are distinct.

At present, two additional HOPP-related manuscripts are under consideration at PLOS ONE. One focuses on waist-to-height ratio (WHtR) as the primary outcome, while the second focuses on metabolic syndrome and cardiometabolic risk factors. The present manuscript differs from both by examining quality of life (QoL), measured using the Inventory of Life Quality in Children and Adolescents (ILC), as the primary outcome.

The distinctions between the manuscripts can be summarized as follows:

Present manuscript (QoL): Examines associations between QoL, moderate-to-vigorous physical activity (MVPA), socioeconomic status (SES), waist-to-height ratio (WHtR), age, and sex over a five-year period.

WHtR manuscript: Examines determinants and longitudinal development of waist-to-height ratio as the primary outcome.

Metabolic syndrome manuscript: Examines metabolic syndrome and cardiometabolic risk indicators as primary outcomes.

Although the manuscripts share the same underlying cohort and partially overlap in study period and selected explanatory variables, they address different research questions, use different primary outcome measures, and lead to different scientific conclusions.

To further clarify:

Study population: Overlapping HOPP cohort.

Study period: Partially or fully overlapping observation period.

Primary outcomes: QoL (present manuscript), WHtR (related manuscript), and metabolic syndrome/cardiometabolic risk (related manuscript).

Research questions: Distinct and non-overlapping.

Statistical analyses: Different analytical models based on different dependent variables and objectives.

Main findings: Different outcome domains and conclusions.

We confirm that the present manuscript represents an independent scientific contribution. No tables, figures, or analyses are duplicated across the manuscripts. Any overlap is limited to the use of the same cohort and selected background variables, which is typical for longitudinal cohort studies and is fully transparent.

We hope this clarification addresses the editor’s concerns and demonstrates that the manuscript constitutes a distinct and independent publication.

Best regards,

Rein Magnus Jensen

On behalf of all authors

Attachment Submitted filename: Response to Reviewers 02.docx

# Editor report: Decision Letter 3

**DOI:** 10.1371/journal.pone.0353686.r007

**Authors:** Bijit Biswas, Bijit Biswas, Bijit Biswas, Bijit Biswas

28 Jun 2026

Physical activity and quality of life in children: Findings from the Health Oriented Pedagogical Project (HOPP)

PONE-D-25-65082R3

Dear Dr. Rein Magnus Jensen,

We’re pleased to inform you that your manuscript has been judged scientifically suitable for publication and will be formally accepted for publication once it meets all outstanding technical requirements.

Within one week, you’ll receive an e-mail detailing the required amendments. When these have been addressed, you’ll receive a formal acceptance letter and your manuscript will be scheduled for publication.

An invoice will be generated when your article is formally accepted. Please note, if your institution has a publishing partnership with PLOS and your article meets the relevant criteria, all or part of your publication costs will be covered. Please make sure your user information is up-to-date by logging into Editorial Manager at Editorial Manager® and clicking the ‘Update My Information' link at the top of the page. For questions related to billing, please contact billing support.

If your institution or institutions have a press office, please notify them about your upcoming paper to help maximize its impact. If they’ll be preparing press materials, please inform our press team as soon as possible -- no later than 48 hours after receiving the formal acceptance. Your manuscript will remain under strict press embargo until 2 pm Eastern Time on the date of publication. For more information, please contact onepress@plos.org.

Kind regards,

Bijit Biswas, MBBS, MD, DNB

Academic Editor

PLOS One

Additional Editor Comments (optional):

Thank you for carefully addressing the comments from the previous review. The manuscript has been improved substantially, and my concerns have been adequately resolved. The study is methodologically sound, the analyses are appropriate, and the conclusions are supported by the data. I have no further comments and recommend acceptance of the manuscript.

Reviewers' comments:

# Editor report: Acceptance letter

**DOI:** 10.1371/journal.pone.0353686.r008

**Authors:** Bijit Biswas, Bijit Biswas, Bijit Biswas, Bijit Biswas

PONE-D-25-65082R3

PLOS One

Dear Dr. Jensen,

I'm pleased to inform you that your manuscript has been deemed suitable for publication in PLOS One. Congratulations! Your manuscript is now being handed over to our production team.

At this stage, our production department will prepare your paper for publication. This includes ensuring the following:

* All references, tables, and figures are properly cited

* All relevant supporting information is included in the manuscript submission,

* There are no issues that prevent the paper from being properly typeset

You will receive further instructions from the production team, including instructions on how to review your proof when it is ready. Please keep in mind that we are working through a large volume of accepted articles, so please give us a few days to review your paper and let you know the next and final steps.

Lastly, if your institution or institutions have a press office, please let them know about your upcoming paper now to help maximize its impact. If they'll be preparing press materials, please inform our press team within the next 48 hours. Your manuscript will remain under strict press embargo until 2 pm Eastern Time on the date of publication. For more information, please contact onepress@plos.org.

You will receive an invoice from PLOS for your publication fee after your manuscript has reached the completed accept phase. If you receive an email requesting payment before acceptance or for any other service, this may be a phishing scheme. Learn how to identify phishing emails and protect your accounts at https://explore.plos.org/phishing.

If we can help with anything else, please email us at customercare@plos.org.

Thank you for submitting your work to PLOS ONE and supporting open access.

Kind regards,

PLOS ONE Editorial Office Staff

on behalf of

Dr. Bijit Biswas

Academic Editor

PLOS One
